# Overexpression of *MpCYS4*, A Phytocystatin Gene from *Malus prunifolia* (Willd.) Borkh., Enhances Stomatal Closure to Confer Drought Tolerance in Transgenic *Arabidopsis* and Apple

**DOI:** 10.3389/fpls.2017.00033

**Published:** 2017-01-24

**Authors:** Yanxiao Tan, Mingjun Li, Yingli Yang, Xun Sun, Na Wang, Bowen Liang, Fengwang Ma

**Affiliations:** State Key Laboratory of Crop Stress Biology for Arid Areas, College of Horticulture, Northwest A&F UniversityYangling, China

**Keywords:** ABA sensitivity, ABA/stress-responsive gene, apple, drought tolerance, plant cystatin, stomatal regulation

## Abstract

Phytocystatins (PhyCys) comprise a group of inhibitors for cysteine proteinases in plants. They play a wide range of important roles in regulating endogenous processes and protecting plants against various environmental stresses, but the underlying mechanisms remain largely unknown. Here, we detailed the biological functions of *MpCYS4*, a member of cystatin genes isolated from *Malus prunifolia*. This gene was activated under water deficit, heat (40°C), exogenous abscisic acid (ABA), or methyl viologen (MV) (Tan et al., 2014a). At cellular level, MpCYS4 protein was found to be localized in the nucleus, cytoplasm, and plasma membrane of onion epidermal cells. Recombinant MpCYS4 cystatin expressed in *Escherichia coli* was purified and it exhibited cysteine protease inhibitor activity. Transgenic overexpression of *MpCYS4* in *Arabidopsis* (*Arabidopsis thaliana*) and apple (*Malus domestica*) led to ABA hypersensitivity and series of ABA-associated phenotypes, such as enhanced ABA-induced stomatal closing, altered expression of many ABA/stress-responsive genes, and enhanced drought tolerance. Taken together, our results demonstrate that *MpCYS4* is involved in ABA-mediated stress signal transduction and confers drought tolerance at least in part by enhancing stomatal closure and up-regulating the transcriptional levels of ABA- and drought-related genes. These findings provide new insights into the molecular mechanisms by which phytocystatins influence plant growth, development, and tolerance to stress.

## Introduction

Cysteine proteinase inhibitors (cystatins) have been identified in many eukaryotes and extensively studied in insects and mammals (Rawlings et al., 2008). Plant cystatins, or phytocystatins (PhyCys), cluster into an independent evolutionary branch on cystatin phylogenetic tree. They are devoid of both putative glycosylation sites and disulfide bridges, and can inactivate catalytic activity of papain-like C1A cysteine proteinases or legumain-like C13 peptidases due to a tight and irreversible interaction (Martinez and Diaz, 2008; Benchabane et al., 2010). Phytocystatins could function in regulating the activity of endogenous cysteine proteinases during a variety of physiological processes, including plant growth and development, the reserves mobilization in seeds and tubers, programmed cell death, and senescence (Belenghi et al., 2003; Martinez et al., 2009; Weeda et al., 2009; Díaz-Mendoza et al., 2014). In addition, they also could inhibit exogenous proteases in the digestive tracts of insects (Outchkourov et al., 2004; Alvarez-Alfageme et al., 2007), protect plants from attacks by pests (Carrillo et al., 2011), and help prevent pathogen invasions (Popovic et al., 2013).

Accumulating evidence suggests that phytocystatins play an important role in plants exposed to stressful environmental conditions (van der Vyver et al., 2003; Zhang et al., 2008; Benchabane et al., 2010; Wang et al., 2012; Sun et al., 2014; Kunert et al., 2015). For example, transgenic tobacco (*Nicotiana tabacum*) plants expressing the rice cystatin, *oryzacystatin-I* (*OC-I*), are more resistant to the negative impacts that chilling stress can have on photosynthesis (van der Vyver et al., 2003). Similarly, Zhang et al. (2008) have shown that overexpression of two cystatins, *AtCYSa* and *AtCYSb*, in transformed *Arabidopsis* (*Arabidopsis thaliana*) is associated with enhanced tolerance to high salt, water deficit, cold, and oxidative stresses, and the amounts of APXb and NADP-ME protein are much higher in transgenic lines. Quain et al. (2014) reported that *OC-I*-inhibited cysteine proteinases could affect plant growth and increase drought stress resistance associated with strigolactone pathways. An *AtCYS4* (*Arabidopsis phytocystatin 4*) transcriptional activator, DREB2C (dehydration-responsive element-binding protein 2C), has been identified that can bind to the *AtCYS4* promoter and regulate the expression of *AtCYS4* in response to heat stress (Je et al., 2014). Although these studies have greatly improved our understanding on the biological roles of PhyCys during plant response to environmental stresses, the underlying mechanisms at the molecular level remain largely unknown.

Plants are sessile organisms capable to adapt diverse adverse growth conditions, including drought, high salinity, heat, extreme light levels, and various metals in the soil. Among them, drought is one of the most serious stresses that affect agricultural production. When plants encounter water deficits, cells trigger a network of signaling events to reprogram their processes. Hormone-mediated signaling pathways, particularly those associated with the synthesis and perception of abscisic acid (ABA), are highly responsive to drought (Knight and Knight, 2001; Bray, 2002). ABA contents in the cells are significantly increased under drought conditions, thereby inducing stomatal closure and gene expression as part of a coping mechanism (Xie et al., 2006; Finkelstein, 2013; Clauw et al., 2015).

Apple (*Malus domestica*), one of the most widely cultivated woody plants in temperate regions, is the fourth most economically important fruit tree after *Citrus* sp., *Vitis vinifera*, and *Musa* sp. (Hummer and Janick, 2009). While, water deficits have become a critical source of abiotic stress that affect apple growth, productivity, and geographic distribution. Therefore, identifying drought-related genes and defining their functions will enrich our knowledge about drought-signaling networks in apple and will be important for improving the adaptability of apple to water-deficit conditions.

We previously identified 26 putative PhyCys genes within the entire apple genome and monitored their transcription patterns in the roots, stems, leaves, flowers, and seeds as well as in response to drought, low temperature (4°C), heat (40°C), ABA, and oxidative stress (Tan et al., 2014a). However, their biological roles had not yet been systematically evaluated. Here, we report that *MpCYS4*, GenBank Accession No. KF477275, original name *MdCYS21*, a stress-responsive cystatin gene isolated from *M. prunifolia*, acts as a positive regulator in drought stress responses, and its function may be due, at least partly, to its interaction within stress-responsive ABA signaling transduction that mediates stomatal regulation and a variety of ABA/stress-related genes expression to cope with drought conditions.

## Materials and methods

### Plant materials and growth conditions

*In vitro* shoot cultures of rootstock M26 from *M. domestica* were sub-cultured at 4-week intervals on an Murashige and Skoog (MS) agar medium containing 0.5 mg L^−1^ indole butyric acid (IBA) and 1.0 mg L^−1^ 6-benzylaminopurine (6-BA). Initial growing conditions were 25°C, 100 μmol photons m^−2^ s^−1^, and a 16-h light/8-h dark photoperiod. For root induction, 4-week-old shoots were shifted to MS medium supplemented with 0.3 mg L^−1^ indoleacetic acid (IAA).

Seeds of *Arabidopsis* ecotype “Columbia” (Col-0) were first incubated in dark at 4°C for 3 days, then surface-sterilized according to the protocol described by Weigel and Glazebrook (2002), and sown on medium containing ½-strength MS salts, sucrose (3%, w/v), and agar (0.75%, w/v). Seedlings were transferred to soil (rich soil:vermiculite, 2:1, v/v) after germination for 7–10 days and grown in a climate chamber at 22°C, 70% relative humidity, 100 μmol photons m^−2^ s^−1^, and 16-h light/8-h dark photoperiod.

### Subcellular localization analysis

For subcellular localization, the coding sequence of *MpCYS4*, without the termination codon, was cloned into the *Xho*I/*Sac*I site of pBI221-GFP vector (Clontech, Palo Alto, CA, USA). The specific primers are shown in Table S1. For transient expression in onion epidermal cells, pBI221-*MpCYS4*-GFP plasmids or blank vector pBI221 were inserted into the cells using a PDS-1000 biolistic helium gun device (Bio-Rad, Hercules, CA, USA), as the protocol described by Diaz et al. (2005). Then, the cells were cultured on MS medium in dark for 24 h before observation of the fluorescent proteins with a LSM510 confocal laser-scanning microscope (Carl Zeiss, Thornwood, NY, USA).

### Cysteine-protease inhibition assay

The coding region of *MpCYS4* was cloned into the *EcoR*V/*Xho*I site of pET-32a expression vector (Novagen, Gibbstown, NJ, USA), introduced into *Escherichia coli* BL21 strain. The specific primers are shown in Table S1. Bacterial cells expressing the fusion protein were harvested after induction with isopropyl β-D-thiogalactopyranoside (IPTG). The recombinant protein was purified using a nickel column chromatography procedure (Novagen) and quantified by method of Bradford (1976). Testing the capacity of the recombinant MpCYS4 protein to inhibit cysteine proteases has been described previously (Gaddour et al., 2001), using papain (EC3.4.22.2; Sigma, St. Louis, MO, USA) and benzoyl-L-arginine-*p*-nitroanilide (BANA) as substrate. Control assays used the equivalent molar amount of purified recombinant protein isolated from *E. coli* that harbored the empty pET-32a vectors. Absorbance of the enzyme reaction was recorded at 405 nm with a benchmark microplate reader (Bio-Rad).

### Transformation and regeneration of *Arabidopsis*

The coding region of *MpCYS4* was cloned into the pBI121 vector (Clontech) under the CaMV 35S promoter. The specific primers are shown in Table S1. Subsequently, the resultant vectors were genetically transformed into *Arabidopsis* ecotype “Col-0.” Transformation was accomplished via the floral dip method using *Agrobacterium tumefaciens* strain EHA105 harboring the recombinant plasmid, as described by Clough and Bent (1998). Putative transgenic *Arabidopsis* plants were selected on MS medium containing 50 mg L^−1^ kanamycin. After further selecting at a 3:1 segregation ratio, T3 homozygous transgenic lines were derived for the phenotypic analysis.

### Seed germination assay

For germination assays, *Arabidopsis* seeds after surface-sterilized, were placed on the germination media containing various concentrations of mannitol (an osmotic agent; Wang et al., 2011) or ABA (A1049; Sigma). Germination was considered complete when radicles had emerged by 1 mm. The experiments were repeated for three times, and approximately 50 seeds per line were sown for each experiment.

### Physiological measurements

During drought period, net photosynthesis (Pn) was monitored using a Li-6400 portable photosynthesis system (LiCor, Huntington Beach, CA, USA), at a constant airflow rate of 500 μmol photons m^−2^ s^−1^, with vapor pressure deficit of 2.0–3.4 kPa and cuvette CO_2_ concentration of 400 μmol CO_2_ mol^−1^ air. Data were obtained from light exposed, fully-expanded leaves on each of five plants. Values for relative water content (RWC) were determined according to method described by Gaxiola et al. (2001). Electrolyte leakage (EL) was determined from leaves as described by Dionisio-Sese and Tobita (1998), with an electrical conductivity meter (DSS-307; SPSIC, Shanghai, China). Chlorophyll was extracted with 80% acetone, and the concentration was determined spectrophotometrically according to the method of Lichtenthaler and Wellburn (1983). ABA was extracted and measured as described by Li et al. (2015), with a high-performance liquid chromatograph (HPLC) (LC2010A; Shimadzu, Kyoto, Japan).

### Stomatal aperture analysis

The ABA-induced stomatal closure was measured from leaves of 4-week-old plants that had been detached and floated abaxial side down on a stomatal opening solution containing 10 mM MES-KOH (pH 6.15), 20 mM KCl, and 1 mM CaCl_2_. After they were incubated for 2 h under lights, they were then treated for 2 h with various concentrations of ABA added to the solution. Obtained from epidermal peels, the stomata were photographed and counted with a JSM-6360LV scanning electronic microscope (JEOL Ltd., Tokyo, Japan) as previously described (Tan et al., 2014b). Their sizes were measured using Image J software, using at least 50 apertures from each treatment.

### Stress tolerance assay in *Arabidopsis*

The rate of transpiration (water loss) was evaluated by placing detached, fresh *Arabidopsis* leaves on open Petri dishes with abaxial side up and weighing them at regular intervals. The treatment was conducted at room temperature. Third to fourth true rosette leaves were collected from 3-week-old soil-grown plants under standard growing conditions as described above. In each experiment, approximately 50 leaves per line were used and the entire test was repeated for three times.

To assess drought resistance of the soil-grown plants, 1-week-old seedlings were transplanted in soil for 3 weeks under standard growing conditions before being subjected to water deficit by withholding water for 14 days (severe stress). The leaf RWC and stomatal apertures were measured from tissues sampled after 7 days. Digital images and plant survival rates were recorded on Day 14. Experimental variations were minimized by growing the same number of plants on each tray. The entire test was repeated at least three times.

For ABA treatment, 100 μM (±)-ABA (Sigma; A1049) was sprayed onto 5-day-old seedlings grown on MS plates to ensure total foliar coverage. In parallel experiments, water-only was sprayed as the control. Samples were collected after 0, 3, 6, 12, or 24 h of treatment, then frozen in liquid nitrogen and stored at −80°C for RNA extractions.

### Genetic transformation and stress tolerance assay in apple

The *MpCYS4* overexpression vector (pBI121-*MpCYS4*) was transferred into apple by *Agrobacterium*-mediated transformation, using leaf discs of apple rootstock M26, as described by Kotoda et al. (2010). After cultivation in dark for 2 weeks, the explants were moved under lights. Their shoots were excised and then propagated on multiplication medium containing 50 mg L^−1^ kanamycin. The rooted plantlets were transferred to plastic pots (4 × 5 × 5 cm) filled with a mixture of forest soil, sand, perlite, and vermiculite. Growth conditions were 24 ± 2°C, 70 ± 5% relative humidity, 100 μmol photons m^−2^ s^−1^, and a 16-h light/8-h dark photoperiod.

After 2 months, the plants were transplanted in plastic culture pots (30 × 26 × 22 cm) contained a mixture of forest soil:organic substrate:sand (5:1:1, v:v:v), and placed in the greenhouse under ambient light, with temperature ranging from 20 to 35°C and a relative humidity of 65–70% at Northwest A&F University, Yangling, China (34°20′N, 108°24′E). After 3 months of growth under this condition, healthy and uniformly sized plants (30 per line, for a total of 60) were subjected to a water deficit. Another 30 plants per line (Control) continued to receive irrigation daily to maintain the saturated soil water content. Soil water content (v/v) was determined with an HH2 Moisture Meter (Delta-T Devices, Cambridge, UK). Drought was induced by withholding water for 8 days. At 9:00 a.m. on alternate days, Pn was recorded on the ninth to twelfth leaves from the stem base of five plants with similar soil water content. Then, the leaves were rapidly frozen in liquid nitrogen and stored at −80°C for RNA extractions. At each time point, another set of samples was collected to determine leaf RWC. After 3 days of treatment, additional leaves were sampled for stomatal observation, ABA measurement, and RNA-Seq analysis. Leaf chlorophyll concentrations and EL values were measured at the final time point.

### RNA extraction, quantification, and gene expression analysis

Total RNA was isolated using E.Z.N.A.® Plant RNA Kit (Omega Biotek, Norcross, GA, USA) according to the manufacturer instructions. RNA integrity was checked on a 1.2% agarose gel and RNA quantity was assessed with a NanoDrop 1000 Spectrophotometer (Thermo Scientific, Wilmington, DE, USA). First-strand cDNAs were synthesized from 2 μg of total RNA with SYBR Prime Script RT-PCR Kit II (TaKaRa, Tokyo, Japan).

Semi-quantitative RT-PCRs were performed to examine the transcript levels of *MpCYS4* in apple and *Arabidopsis*. Apple *Actin* and *Arabidopsis Actin* were used as respective loading controls. Quantitative real-time PCR (qRT-PCR) was conducted on an iQ5.0 detection instrument (Bio-Rad) using SYBR Green qPCR kits (TaKaRa). Reactions were performed in triplicate with a volume of 20 μL, and apple *EF-1*α or *Arabidopsis Actin* was amplified as the appropriate internal control. The relative quantity of target gene transcript was determined by applying the 2^−ΔΔCT^ method (Livak and Schmittgen, 2001). Primers are listed in Tables S1, S2. Each value was expressed as mean and standard deviation (SD) calculated from the result of three independent replicates.

### RNA-seq analysis

Transcriptional profiling of WT apple and transgenic line #4 was conducted by RNA-Seq. Three biological replicates were used for each genotype, with samples collected from plants grown under normal conditions as well as after drought induced by withholding irrigation for 3 days. Each biological replicate comprised samples from three to four plants that were mixed after collection to generate one independent pool. Total RNA (2 μg per sample) was prepared and used for both cDNA library construction (Zhong et al., 2011) and RNA-Seq by the Gene Denovo Biotechnology Corporation (Guangzhou, China), with an Illumina HiSeq™ 2500 (Illumina, San Diego, CA, USA). The files of raw fastq were checked by FastQC (http://www.bioinformatics.babraham.ac.uk/projects/fastqc). After the adaptor sequences were removed using fastqmcf (https://code.google.com/p/ea-utils/wiki/FastqMcf), the reads were aligned to the database for *M. domestica* genome (Velasco et al., 2010; http://www.nature.com/ng/journal/v42/n10/full/ng.654.html) using *TopHat2* (Kim et al., 2013). All usable reads were then normalized into fragments per kilobase of transcript per million mapped reads (FPKM) (Mortazavi et al., 2008). The transcript abundance differences among samples were computed with the ratio of FPKM values. The significance of those differences was evaluated with a false discovery rate (FDR) as the threshold of *P*-value for multiple tests (Yekutieli and Benjamini, 2001). Genes with FDR <0.05 and fold-changes >2 were used in later analyses. Our methods for assessing the significance of differentially expressed genes (DEGs) followed those described by Anders and Huber (2010).

### Statistical analysis

All statistical analyses were conducted using SigmaPlot software (Systat Software). Data were analyzed via one-way ANOVA using the SPSS-11. Statistical differences were compared based on Student's *t*-tests. ^*^, ^**^, and ^***^ indicates significance level at *P* < 0.05, *P* < 0.01, and *P* < 0.001, respectively.

## Results

### Molecular characterization, subcellular localization, and cysteine-protease inhibition of *MpCYS4*

**Figure 1:**
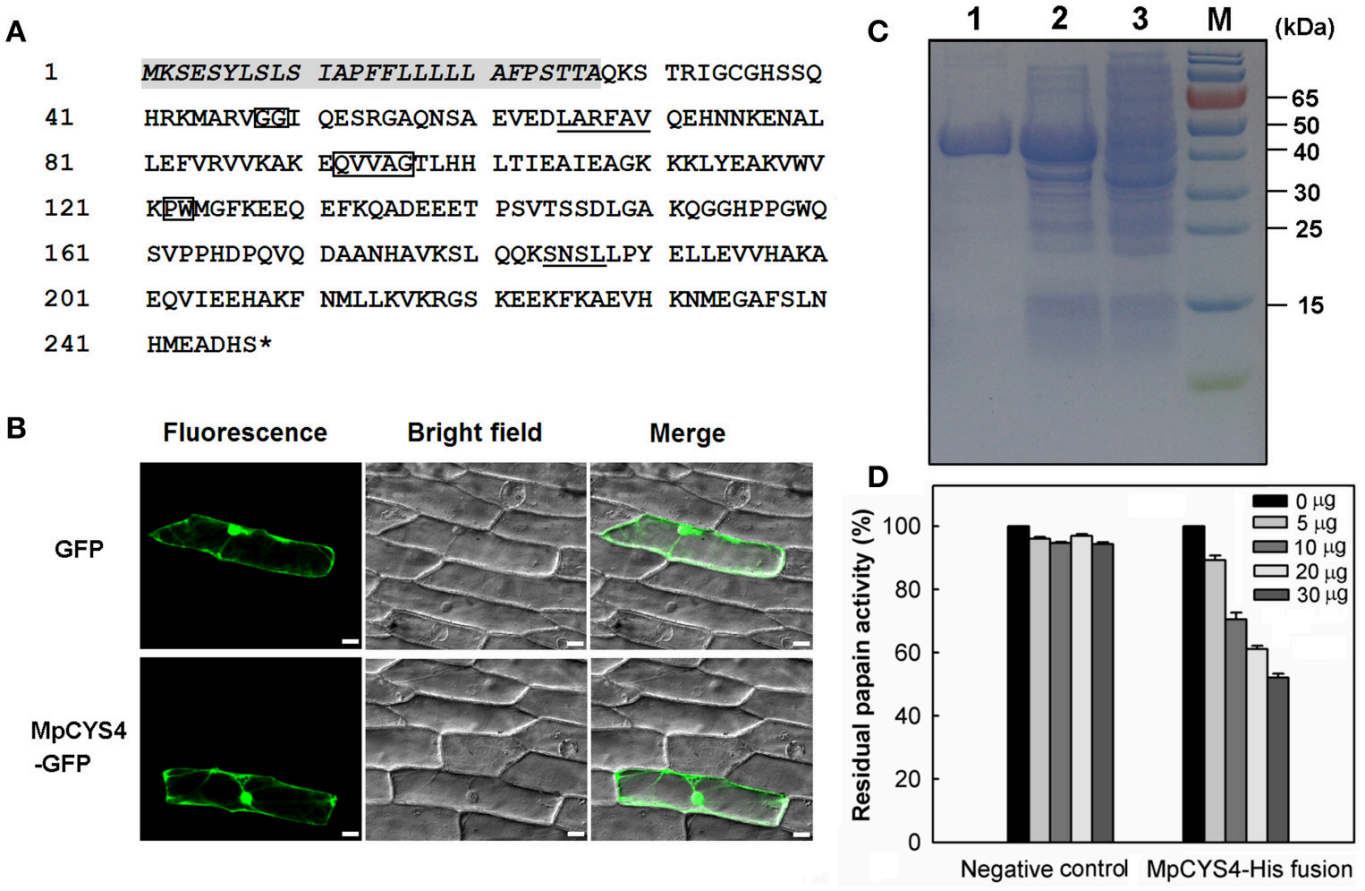
**Amino acid sequence analysis, subcellular localization, and cysteine-protease inhibition of MpCYS4. (A)** Deduced amino acid sequence of MpCYS4, with putative signal peptide italicized. Conserved signature sequences of PhyCys are enclosed with rectangles (N-terminal G; QXVXG; P/AW); LARFAV-like motif and SNSL motif are underlined. ^*^Represents the termination codon; **(B)** Subcellular localization of MpCYS4–GFP fusion protein in onion epidermal cells. GFP alone or MpCYS4–GFP, corresponding bright field images, and overlay from merging of bright and fluorescent illumination are shown. GFP or MpCYS4–GFP fusion was driven by CaMV 35S promoter. Onion epidermal peels were bombarded with DNA-coated gold particles, and GFP expression was visualized 24 h later. Scale bar, 50 μm; **(C)** SDS-PAGE (12%) analysis of bacterial expression and purification of MpCYS4 representing (1) purified recombinant MpCYS4–His fusion protein, (2) total soluble protein fraction after 5 h of IPTG induction, and (3) total soluble protein fraction without IPTG induction; **(D)** *In vitro* inhibition of cysteine protease activity by recombinant MpCYS4 protein. Inhibition of recombinant MpCYS4 protein to papain expressed as residual enzyme activity in presence of increasing inhibitor concentrations. Purified induced protein of empty pET32a was used as negative control. Data are means ± SD from 3 independent experiments.

*MpCYS4* encoded a phytocystatin protein containing 248 amino acids with a predicted molecular weight of 27.72 kDa. Characteristic sequences and motifs of MpCYS4 are highlighted in Figure 1A, including the reactive site QVVAG in the central part, two glycine residues (GG) near the N-terminal, PW residues near the C-terminal, and a SNSL motif in the C-terminal extension. The typical sequence of PhyCys, LARFAV, was also localized at the N-terminus of the protein.

To investigate the subcellular localization of MpCYS4 protein, we fused it with GFP at the N-terminal and transiently expressed in onion epidermal cells. After 24 h, we detected the accumulation of MpCYS4-GFP fusion protein in the cell membrane, cytoplasm, and nucleus of those cells, which was the same as the subcellular localization of the GFP protein (Figure 1B). This result was consistent with previous reports for other cystatins, such as BvM14-cystatin from *Beta vulgaris* “M14” (Wang et al., 2012) and GsCPI14 from *Glycine soja* (Sun et al., 2014).

His-tagged fusion protein of MpCYS4 was expressed in *E. coli* cells and affinity-purified (Figure 1C). To confirm that this gene is a functional cysteine protease inhibitor, we used an *in vitro* assay with papain as substrate. Testing the behavior of the recombinant MpCYS4 protein against cysteine protease revealed that papain activity was blocked in a concentration-dependent manner (Figure 1D). Meanwhile, when the purified and induced protein of empty pET32a was added into the reaction as a negative control, this activity was not inhibited.

### *MpCYS4* overexpression enhanced the drought tolerance of *Arabidopsis*

We previously demonstrated that *MpCYS4* is up-regulated by drought, heat, exogenous ABA, or MV treatments in leaves of *M. prunifolia* (Tan et al., 2014a). We further investigated its potential functioning during abiotic stress responses by analyzing the phenotypes of *MpCYS4*-overexpressing plants. The coding sequence of *MpCYS4* was inserted into a transgenic vector and constitutively expressed in transgenic *Arabidopsis* plants. In all, we obtained 16 independent T3 homozygous lines and verified them via PCR analysis (data not shown). Two representative lines with the highest expression—overexpressing (OE)-4 and OE-13—were selected for further examination (Figures S1A,B). On a MS growth medium, seed germination and elongation of the primary roots from *P*_*CaMV*35*S*_*:MpCYS4* was retarded when compared with the wild-type (WT) (Figures S1C–E), while no obvious difference was observed for soil-grown plants in their aerial parts.

Because *MpCYS4* expression was strongly induced by water deficit in *M. prunifolia* (Tan et al., 2014a), our study focused on the drought tolerance of 35S:*MpCYS4* transgenic *Arabidopsis*. Seeds from the 35S:*MpCYS4* transgenic and WT plants were germinated and seedlings were then grown on a MS medium containing 400 mM mannitol. Both germination and post-germinative growth of all genotypes were repressed, albeit to varying degrees. For example, the seeds of transgenic lines (OE-4 and OE-13) germinated much faster compared to the WT. After 14 days, only 19% of the latter had germinated compared with 83% for OE-4 and 73% for OE-13 (Figure S1C). Seedlings of the transgenic lines also grew faster and had longer primary roots and higher fresh weights (FWs) than the WT (Figures S1D,E). The results suggested that *MpCYS4* overexpression alleviates the adverse effects induced by osmotic stress on *Arabidopsis* growth.

**Figure 2:**
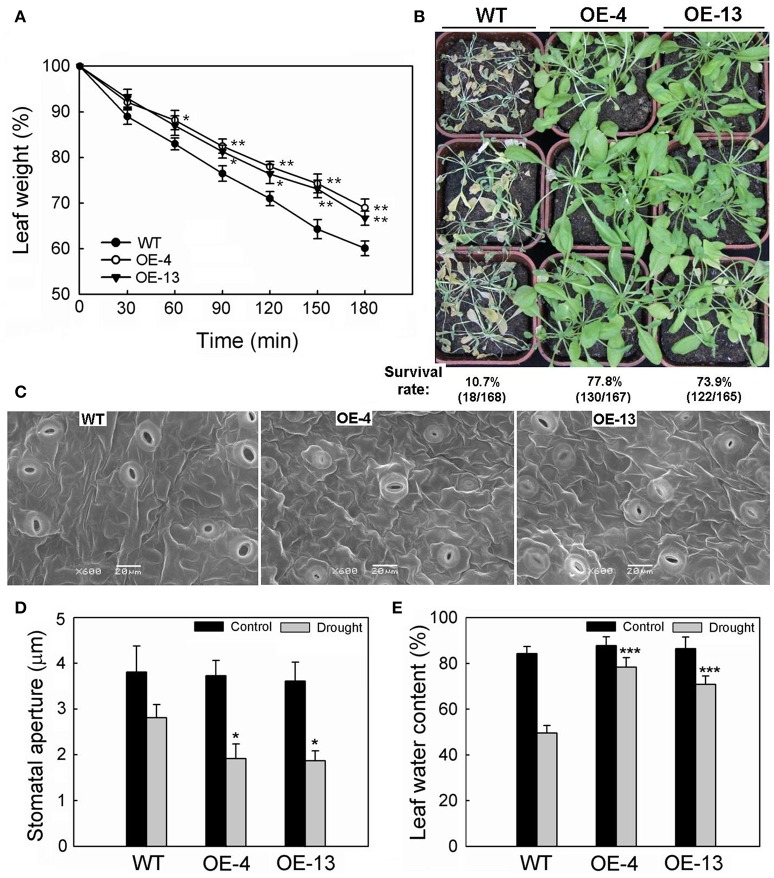
**Responses to drought by 35S:*MpCYS4* and wild-type (WT) *Arabidopsis*. (A)** Transpiration rates. Leaves at same developmental stages were excised and weighed at various time points after detachment. Data are means ± SD from 3 independent experiments. Values were significantly different from WT at ^*^P < 0.05 or ^**^P < 0.01, based on Student's *t*-tests; **(B)** Drought tolerance test. Watering of 4-week-old plants was stopped for 14 days. Survival rates (% indicated below each line) were calculated as number of surviving plants divided by total number of plants tested in 3 independent experiments; **(C)** Stomatal apertures of WT (left), 35S:*MpCYS4* transgenic plants OE-4 (center), and OE-13 (right). Stomatal guard cells were observed during middle of water-deficit period via scanning electron microscopy. Scale bar, 20 μm; **(D)** Measurement of stomatal aperture on WT and transgenic (OE-4 and OE-13) plants corresponding to **(C)**. Data are mean ratios from 3 independent experiments. At least 50 stomatal apertures were measured per line per experiment. ^*^Values were significantly different from WT at *P* < 0.05, based on Student's *t*-tests. **(E)** Relative water content in WT and transgenic lines OE-4 and OE-13 after 7 days of drought treatment. Data are means ± SD from 3 independent experiments. ^***^Values were significantly different from WT at *P* < 0.001, based on Student's *t*-tests.

The rate of water loss was slower for 35S:*MpCYS4* plants than the WT, based on FW measurements from detached rosette leaves. As shown in Figure 2A, after 3 h on open Petri dishes, the FWs of detached rosette leaves were reduced to 69% of the original value for OE-4, 66.7% for OE-13, and 60.1% for the WT. In addition, the survival rates for 35S:*MpCYS4* plants deprived of water for 14 days were higher (77.8% for OE-4, 73.9% for OE-13) than for similarly treated WT plants (10.7%) (Figure 2B). These results confirmed that overexpression of *MpCYS4* could greatly improve drought resistance in transgenic *Arabidopsis*.

### *MpCYS4* affected stomatal behavior in response to drought stress

The function of *MpCYS4* in controlling water losses prompted our investigation into the operation of stomatal behavior, a major factor affecting water-holding capacity in plant leaves. The stomatal density did not show significant difference between 35S:*MpCYS4 Arabidopsis* and WT plants before and after 7 days of drought treatment (Figure S2), which suggested that the higher drought resistance of 35S:*MpCYS4 Arabidopsis* is unrelated to stomatal density. Whereas, compared with the WT, stomatal apertures were smaller on 35S:*MpCYS4* leaves when subjected to water-deficit conditions (Figure 2C). As shown in Figure 2D, no obvious difference was detected among 35S:*MpCYS4* and wild-type plants under normal conditions. However, drought treatment led to a higher occurrence of closure in the transgenic lines, with stomatal dimensions for the WT being reduced to approximately 74% of the size measured from untreated plants vs. reductions of 51.5 and 51.8% of the original, unstressed sizes for transgenic lines OE-4 and OE-13, respectively. Consistent with these results, the RWC of leaves in the WT was 49.6%, as opposed to 78.4% for OE-4 and 70.9% for OE-13 after 7 days of drought treatment (Figure 2E). These findings indicated that, during periods of dehydration, the stomata of the transgenic lines responded to water deficits better than did the WT plants.

### Transgenic *Arabidopsis* plants over-expressing *MpCYS4* were hypersensitive to abscisic acid

**Figure 3:**
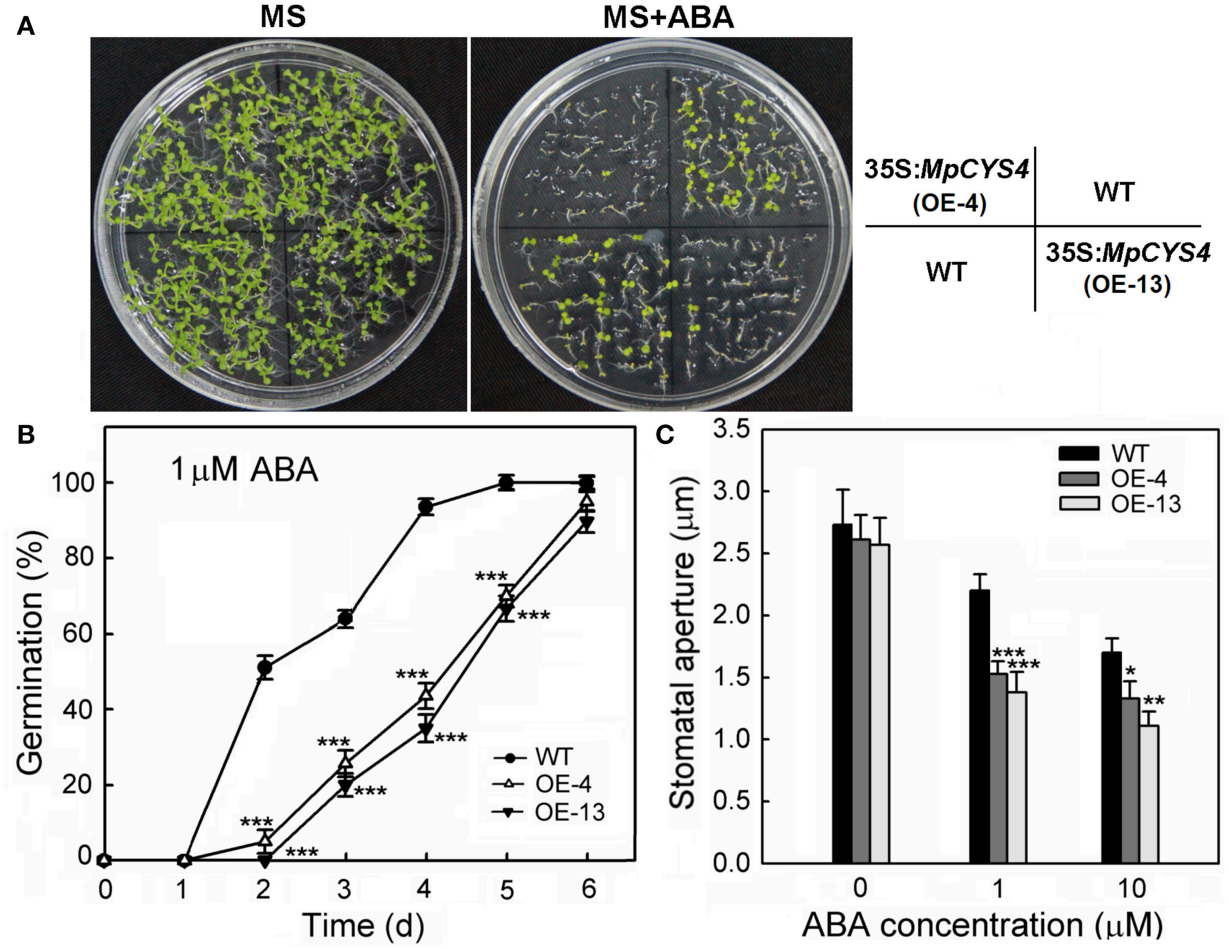
**Responses of wild-type (WT) and 35S:*MpCYS4 Arabidopsis* upon ABA treatment. (A)** ABA sensitivity in seedlings. Approximately 50 seeds from 4 independent seed lots of simultaneously grown WT and 35S:*MpCYS4* (lines OE-4 and OE-13) were surface-sterilized and sown on 1/2 Murashige and Skoog (MS) agar plates supplemented with ABA (mixed isomers; Sigma A1049). Images were taken on Day 9 after germination on agar medium supplemented with 0 μM or 0.25 μM ABA; **(B)** Germination rates after 6 days. Seeds were surface-sterilized and sown on 1/2 MS agar plates with 1 μM ABA. Results are means ± SD from 3 independent experiments (approximately 50 seeds per line per experiment); **(C)** Size of stomatal apertures. Stomata were fully opened prior to ABA treatment. Rosette leaves of 4-week-old plants were detached and floated abaxial side down on opening solution for 2 h prior to ABA treatment. Leaves were then treated with 0, 1, or 10 μM ABA for 2 h before apertures were measured. Stomatal apertures in epidermal peels were observed under scanning electronic microscope. Data are mean ratios ± SD from 3 independent experiments. At least 50 stomatal apertures were measured per treatment. Values were significantly different from WT at ^*^P < 0.05, ^**^P < 0.01, or ^***^P < 0.001, based on Student's *t*-tests.

Stomatal closure is one of the most important ABA-induced environmental responses. Then, we examined 35S:*MpCYS4* plants to determine whether *MpCYS4* overexpression affects ABA sensitivity. When germinated on an MS medium supplemented with ABA, both the germination and post-germinative developmental stages of the 35S:*MpCYS4* proved hyposensitive to exogenous ABA compared with the WT (Figures 3A,B). In the presence of 0.25 μM ABA, 35S:*MpCYS4* seedlings were dramatically restrained while the WT seedlings showed expanded, green cotyledons (Figure 3A). To determine whether *MpCYS4* is involved in ABA-induced stomatal movement, we treated WT and 35S:*MpCYS4* leaves with ABA and found that such treatment resulted in a higher degree of closure in the transgenic plants (Figure 3C). Thus, *MpCYS4* seems to have an important role in ABA-mediated control of the guard cells.

### Constitutive expression of *MpCYS4* affected expression of ABA-responsive genes

Our germination and post-germination assays revealed an increased sensitivity by 35S:*MpCYS4* plants under ABA exposure, while endogenous ABA levels did not show significant difference between 35S:*MpCYS4 Arabidopsis* and WT seedlings before and after subjected to 100 μM ABA for 24 h (Figure S3). The specific and dramatic induction of expression for stress genes represented a prominent response to ABA at the molecular level. Therefore, we used 35S:*MpCYS4* and WT plants treated with 100 μM ABA to assess the expression patterns of several ABA-responsive genes by qRT-PCR.

**Figure 4:**
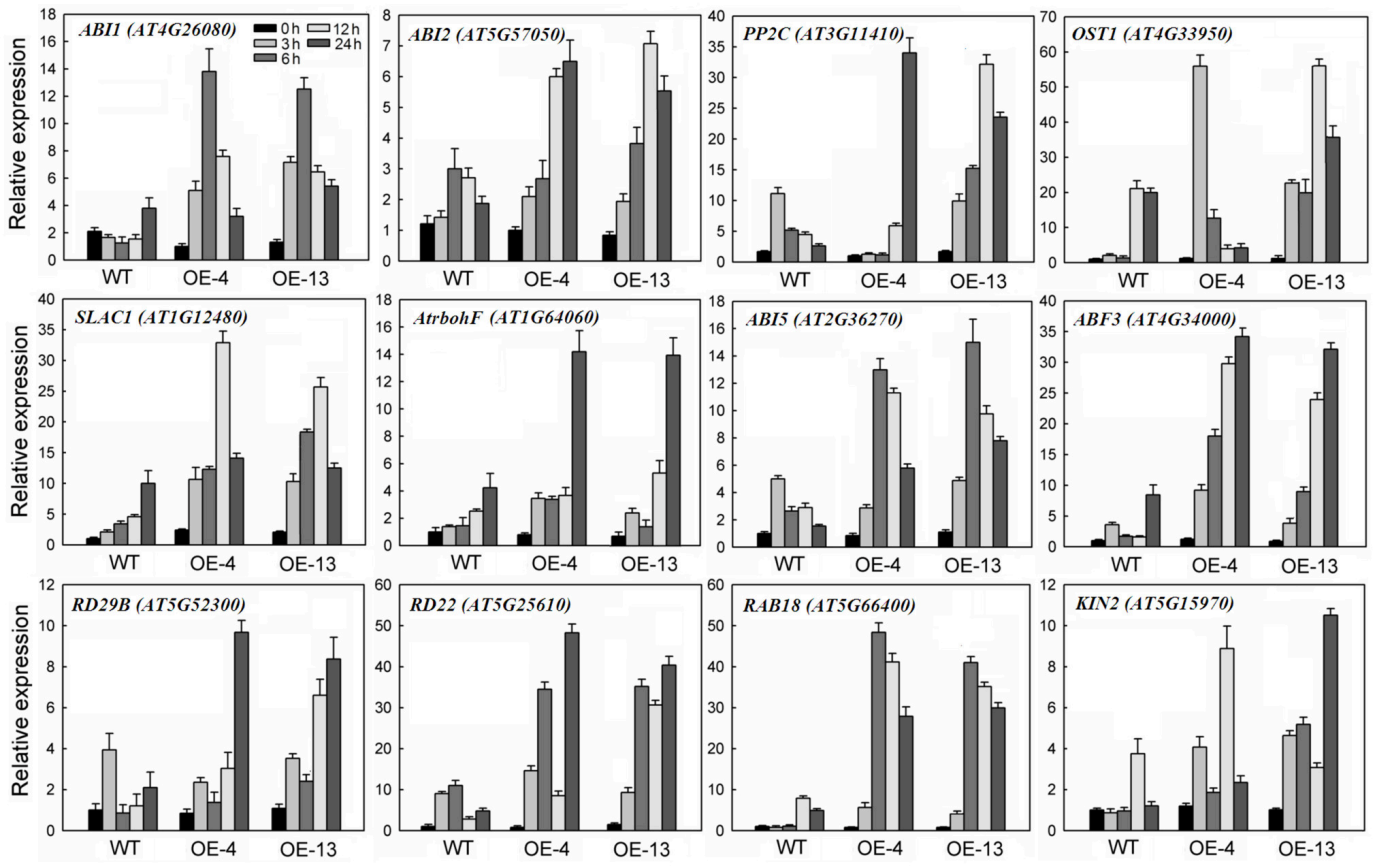
**Quantitative real-time PCR analysis of expression of ABA- and stress-responsive genes in wild-type (WT) and 35S:*MpCYS4* transgenic *Arabidopsis*, as induced by 100 μM ABA**. All transcript levels were normalized relative to WT under non-treated control (0 h) conditions. *AtActin* served as reference gene. Data are means ± SD of 3 independent experiments.

Upon ABA treatment, *three* A-type protein phosphatase 2C (PP2C) family members—*ABA-INSENSITIVE1* (*ABI1*), *ABI2*, and *AtPP2C*—were induced in the WT and even more strongly in 35S:*MpCYS4* seedlings (Figure 4). Other genes in the molecular network of the ABA signaling pathway were also tested, e.g., *OPEN STOMATA1* (*OST1*); the NADPH oxidase F (*AtrbohF*); the anion channel *SLOW ANION CHANNEL-ASSOCIATED 1* (*SLAC1*); ABA-responsive promoter element (ABRE) binding transcription factors *ABI5* and *ABF3*; and other downstream targets such as *LOW TEMPERATURE-INDUCED 65-kDa PROTEIN* (*RD29B*), *RESPONSIVE TO ABA 18* (*RAB18*), *RESPONSIVE TO DEHYDRATION 22* (*RD22*), and *COLD-REGULATED PROTEIN COR6.6* (*KIN2*), which serve as marker genes for monitoring ABA and stress responses in plants. In our experiments, those genes were highly induced upon ABA treatment in the WT plants and even significantly more in the 35S:*MpCYS4* plants. Although expression was detected for all of them, the extent and kinetics of this induction differed among markers. These findings suggested that *MpCYS4* could interact with ABA-mediated signaling process and affect multiple ABA-responsive genes.

### *MpCYS4* overexpression enhanced drought tolerance of transgenic apple and promoted stomatal closure in response to drought stress

**Figure 5:**
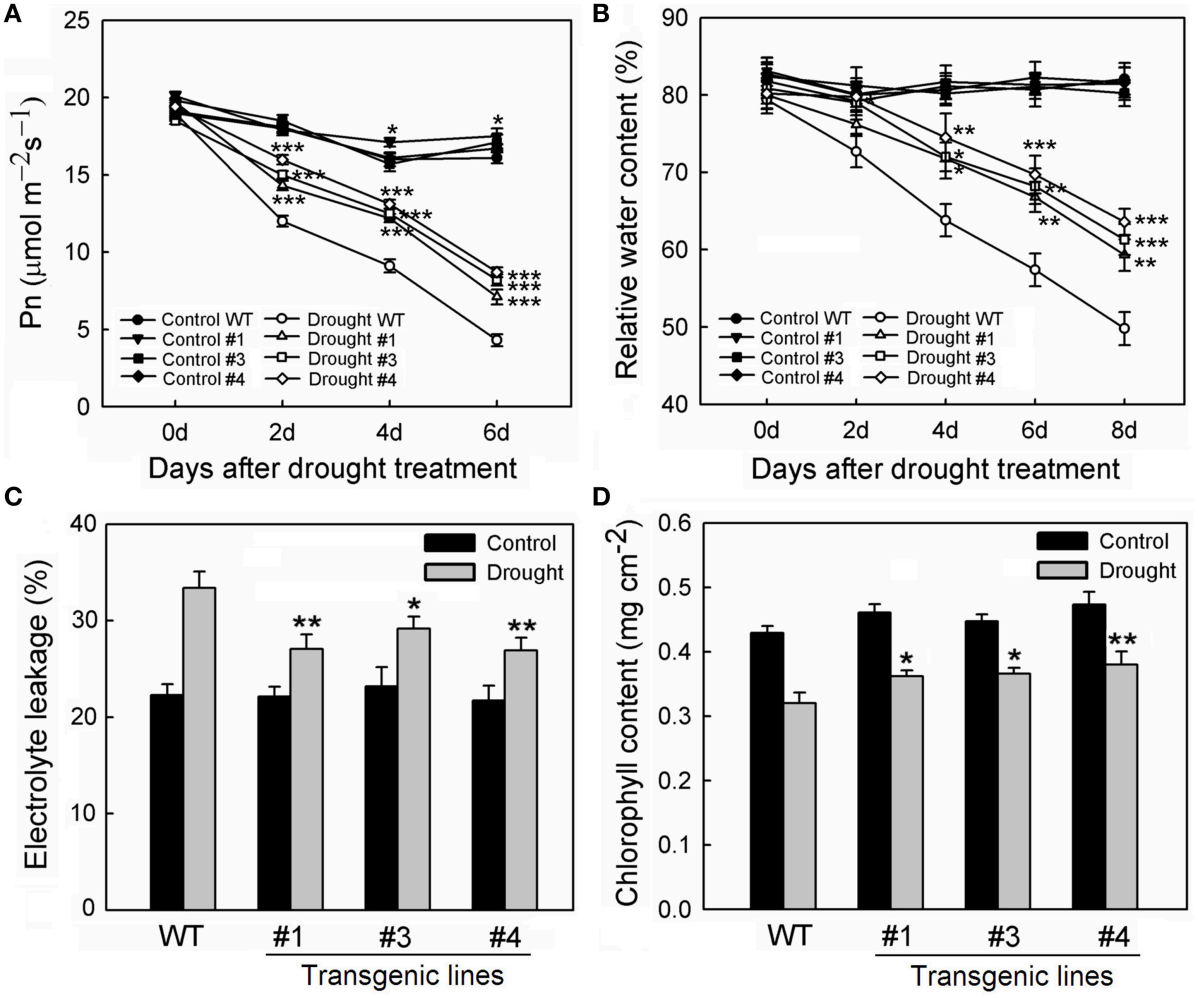
**Comparison of drought tolerance among wild-type (WT) and 35S:*MpCYS4* transgenic apple lines #1, #3, and #4**. Plants of all genotypes were subjected to drought by withholding water for 8 days. Net photosynthetic rate (Pn) **(A)** and relative water content **(B)** were measured on alternate days; electrolyte leakage **(C)** and chlorophyll concentration **(D)** were measured on Day 8 of treatment. Results are means ± SD from 3 independent experiments. Values were significantly different from WT at ^*^P < 0.05, ^**^P < 0.01 or ^***^P < 0.001, based on Student's *t*-tests.

To examine whether *MpCYS4* confers tolerance to drought in apple, *MpCYS4* transgenic apples were obtained. The three *MpCYS4*-overexpressing lines—#1, #3, and #4—which were detected with higher *MpCYS4* transcript level were chosen for further investigation (Figures S4A,B). Under normal growing conditions, the aerial portions of those transgenic plants showed apparent dwarfing and slower development when compared with the traits of the WT (Figures S4C–F). However, the root systems did not show obviously different among the transgenic lines and the WT (data not shown). Drought stress was induced by withholding water from these plants for 8 days. No obvious difference was observed among the tested lines on their growth potential at the onset of the experiment. While, the transgenic lines grew much better than the WT as the stress period was prolonged. The rate of net photosynthesis decreased rapidly after drought treatment, with rates being significantly lower for the WT than for 35S:*MpCYS4* plants (Figure 5A). In addition, the RWC calculated for leaves from each line was significantly reduced under drought conditions. However, overexpression of *MpCYS4* alleviated this response obviously over time (Figure 5B). Consistently, this phenotype was also confirmed by other measurements, with the transgenic lines having much less EL and higher chlorophyll concentrations than the WT at the end of the treatment period (Figures 5C,D).

**Figure 6:**
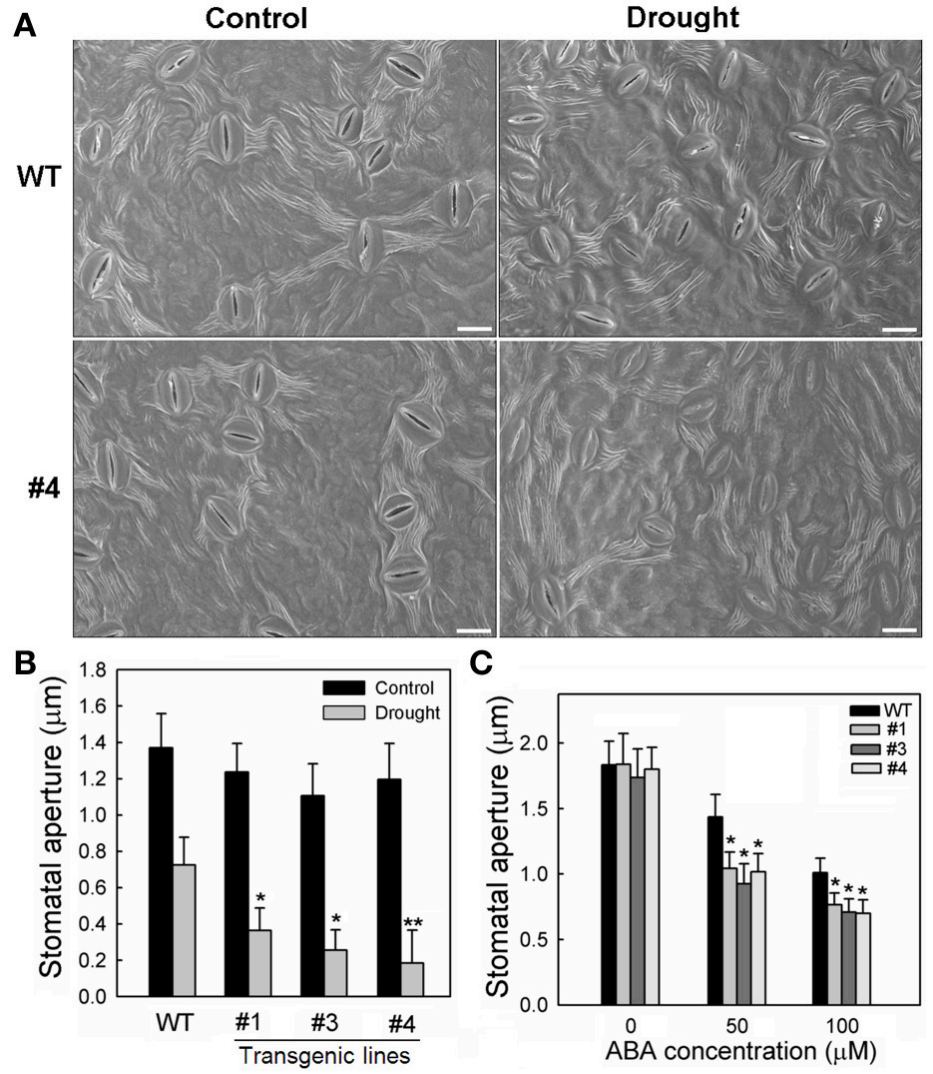
**Stomatal responses by wild-type (WT) and 35S:*MpCYS4* transgenic apple lines #1, #3, and #4 under drought or ABA treatment. (A)** Stomatal guard cells of WT and transgenic lines were observed on Day 3 of water-deficit period via scanning electron microscopy. Representative photographs for stomata from WT and #4. Scale bar, 20 μm; **(B)** Quantitative data for stomatal apertures on leaves from tested plants; **(C)** Effects of ABA treatment on apertures of WT and *MpCYS4*-overexpressing lines #1, #3, and #4. Data are mean ratios ± SD of 3 independent experiments. At least 50 stomatal apertures were measured per line per experiment. Values were significantly different from WT at ^*^P < 0.05 or ^**^P < 0.01, based on Student's *t*-tests.

Meanwhile, the stomata characters of the 35S:*MpCYS4* plants and WT plants were checked using scanning electron microscopy after water was withheld for 3 days. Under normal conditions, both the stomatal density and aperture sizes did not differ significantly between the two genotypes (Figure S5; Figures 6A,B). However, the transgenic lines showed much greater stomatal closure than the WT when drought stress was induced (Figure 6B). Stomatal apertures were also measured from overexpressing and WT plants in response to ABA treatment. In the absence of ABA, sizes were not obviously different among genotypes. However, after 2 h of exposure to 50 or 100 μM ABA, the degree of closure was greater in the 35S:*MpCYS4* plants (Figure 6C), thereby indicating that they had a drought-insensitive phenotype associated with ABA-mediated stomatal closure under the water deficit. Then we examined 35S:*MpCYS4* apple plants to determine whether *MpCYS4* overexpression affects endogenous ABA levels. As shown in Figure S6, no obvious difference was detected among 35S:*MpCYS4* and WT plants under normal conditions. Drought stress increased the levels of ABA in both WT and *MpCYS4* transgenic lines, but no significant differences appeared among them. These data indicated that *MpCYS4* could function in plants ABA sensitivity but not in the biosynthesis of ABA under drought stress conditions.

### *MpCYS4* overexpression led to dramatic transcriptomic alterations in apple leaves

To obtain further insight into the molecular mechanisms by which *MpCYS4* mediates tolerance to drought stress in apple, we performed a large-scale RNA sequencing (RNA-Seq) with RNA extracts from leaves of WT and #4 transgenic plants either grown under normal conditions or subjected to drought by withholding irrigation for 3 days. A total of 12 samples were used with three biological replicates for each genotype. Clean reads were obtained after removing rRNA contaminated reads, with mapping to the genome sequence made up approximately 70% (Table S3). To further evaluate the robustness of the RNA-Seq data, we calculated the correlation coefficients of the transcriptome profiles among the 12 samples. The value could reach 0.99 between each set of biological replicates (Table S4). Based on a two-fold criterion, 82 and 69 genes were up and down-regulated, respectively, in the transgenic line when compared with the WT in the absence of drought stress (Figure S7A). Under water deficit, even more genes exhibited differential expression in line #4 than in WT, with 1556 and 1327 genes being up and down-regulated, respectively (Figure S7B). Among the DEGs, 15 and 8 were commonly up or down-regulated, respectively, in #4 under control and after water was withheld for 3 days (Figure S7C).

**Figure 7:**
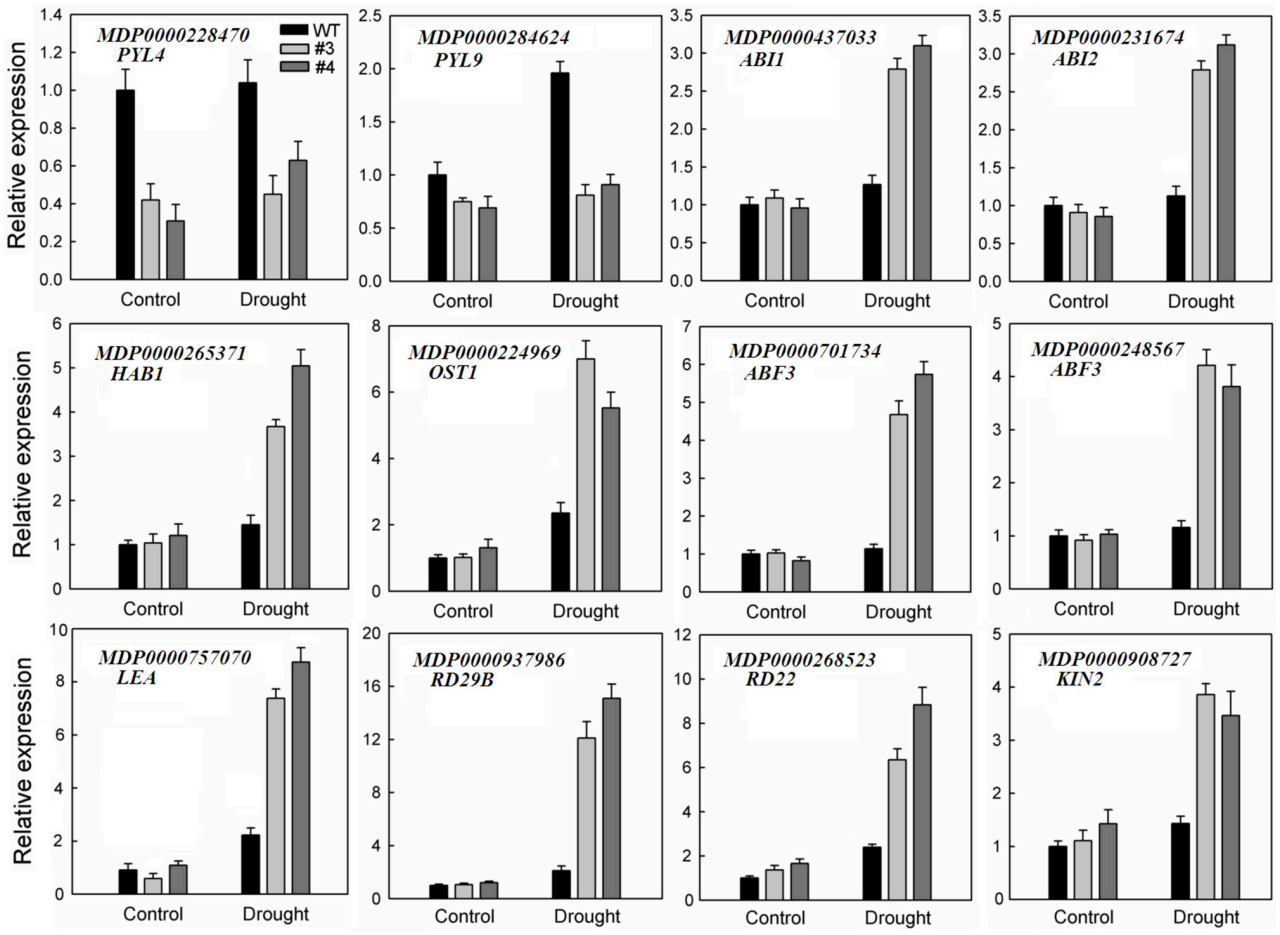
**Validation via quantitative real-time RT-PCR analysis of selected transcriptome-based ABA and stress-responsive differentially expressed genes (DEGs) in 35S:*MpCYS4* transgenic apple compared with wild-type (WT) under drought conditions**. All transcript levels were normalized relative to WT for non-treated control. *MdEF-1*α served as reference gene. Data are means ± SD of 3 independent experiments.

It is worth mentioning that many DEGs involved in ABA signaling transduction and stress response were identified under water-deficit conditions (Table 1), including *PYL4* (MDP0000228470) and *PYL9* (MDP0000284624), which were down-regulated in stressed transgenic line. By contrast, genes encoding *ABI1* (MDP0000437033), *ABI2* (MDP0000231674), *HAB1* (MDP0000265371 and MDP0000178692), *OST1* (MDP0000224969), and the ABRE binding transcription factor *ABF3* (MDP0000701734 and MDP0000248567) were up-regulated in those transgenic plants. Furthermore, transcript levels of several other regulatory proteins associated with stress signal transduction, such as mitogen-activated protein kinase, calcium-dependent protein kinase and transcription factors, including NAC, WRKY, MYB, MYC, MADS, DREB, basic helix-loop-helix (bHLH) transcription factor, heat stress transcription factor (HSF), and APETALA2/ethylene-responsive factor (AP2/ERF) were activated in the overexpression line. In addition, the genes encoding major stress-responsive functional proteins that have been demonstrated to play a direct role in stress response, such as ascorbate peroxidase (APX), catalase (CAT), glutathione S-transferase (GST), late embryogenesis abundant protein (LEA), RD29B, RD22, and KIN2 were also enriched in drought-treated 35S:*MpCYS4* plants. To validate the results from this RNA-Seq analysis, we used qRT-PCR to evaluate the expression of 12 of those genes (Figure 7). It was evident that the expression patterns of those selected genes were largely consistent with what we found from our RNA-Seq data despite the difference in absolute fold-changes between the two methods. This demonstrated that our RNA-Seq results were reliable.

**Table 1:** **Differentially expressed genes (DEGs) involved in ABA signaling transduction and stress response in the transgenic apple compared to wild-type under drought stress**.

**Apple genes identification^a^**	***Arabidopsis*** **homolog^b^**	**Annotation**	**Fold change^c^**	***P-*****value**
MDP0000228470	AT2G38310	Abscisic acid receptor PYL4	−3.15	1.49E-09
MDP0000284624	AT1G01360	Abscisic acid receptor PYL9	−2.43	9.41E-18
MDP0000437033	AT4G26080	Protein phosphatase 2C 56 (ABI1)	2.89	1.57E-07
MDP0000231674	AT5G57050	Protein phosphatase 2C 77 (ABI2)	2.17	5.12E-05
MDP0000265371	AT1G72770	Protein phosphatase 2C 16 (HAB1)	2.35	0.00014
MDP0000178692	AT1G72770	Protein phosphatase 2C 16 (HAB1)	2.28	0.00076
MDP0000296566	AT2G29380	Highly ABA-induced PP2C protein 3 (HAI3)	8.29	3.84E-14
MDP0000224969	AT4G33950	Serine/threonine-protein kinase OST1	2.29	0.00075
MDP0000701734	AT4G34000	Abscisic acid responsive elements-binding factor 3	2.39	9.46E-06
MDP0000248567	AT4G34000	Abscisic acid responsive elements-binding factor 3	2.79	3.01E-17
MDP0000212585	AT4G08500	Mitogen-activated protein kinase kinase kinase 1	2.28	4.16E-07
MDP0000868064	AT5G66850	Mitogen-activated protein kinase kinase kinase 5	12.7	0.00072
MDP0000220179	AT5G66850	Mitogen-activated protein kinase kinase kinase 5	2.12	5.01E-06
MDP0000593502	AT1G07880	Mitogen-activated protein kinase 13	3.08	1.84E-17
MDP0000297184	AT4G35310	Calcium-dependent protein kinase 5	2.98	4.34E-07
MDP0000480581	AT1G61110	NAC transcription factor family protein NAC025	3.11	1.43E-05
MDP0000481448	AT1G61110	NAC transcription factor family protein NAC025	3.50	0.004387
MDP0000868556	AT1G61110	NAC transcription factor family protein NAC025	3.04	3.63E-07
MDP0000262990	AT2G17040	NAC transcription factor family protein NAC036	5.58	0.00381
MDP0000324718	AT3G15210	Ethylene-responsive transcription factor 4	2.87	1.16E-16
MDP0000923579	AT5G47230	Ethylene-responsive transcription factor 5	2.43	0.00029
MDP0000258562	AT3G50260	Ethylene-responsive transcription factor ERF011	6.07	0.00174
MDP0000218344	AT2G20880	Ethylene-responsive transcription factor ERF053	4.11	1.46E-07
MDP0000764803	AT2G20880	Ethylene-responsive transcription factor ERF053	14.18	4.53E-28
MDP0000683814	AT4G39780	Ethylene-responsive transcription factor ERF060	3.38	3.47E-33
MDP0000453797	AT4G39780	Ethylene-responsive transcription factor ERF060	2.65	2.35E-13
MDP0000175375	AT4G34410	Ethylene-responsive transcription factor ERF109	6.38	0.00017
MDP0000185288	AT4G31550	WRKY transcription factor 11	2.42	0.00045
MDP0000307516	AT1G80840	WRKY transcription factor 40	2.40	0.00012
MDP0000299114	AT4G23810	WRKY transcription factor 53	2.43	2.77E-07
MDP0000219647	AT4G23810	WRKY transcription factor 53	8.13	0.00086
MDP0000123888	AT5G13080	WRKY transcription factor 75	3.56	0.00031
MDP0000198054	AT5G51990	Dehydration-responsive element-binding protein 1D	10.57	5.68E-05
MDP0000165880	AT5G05410	Dehydration-responsive element-binding protein 2A	8.04	1.81E-30
MDP0000275800	AT5G67300	Transcription factor MYB44	2.40	0.00025
MDP0000210970	AT1G14350	MYB transcription factor FLP	44.99	0.00018
MDP0000259898	AT1G68320	R2R3-MYB transcription family (MYB62)	7.34	4.86E-05
MDP0000782908	AT4G17880	Transcription factor MYC4	2.82	2.58E-13
MDP0000119495	AT4G17880	Transcription factor MYC4	15.46	8.73E-07
MDP0000262020	AT1G27660	Transcription factor bHLH110	7.44	0.00138
MDP0000296508	AT3G20640	Transcription factor bHLH123	4.46	6.05E-07
MDP0000232313	AT4G24540	MADS-box protein AGL24	10.11	1.83E-60
MDP0000322567	AT4G24540	MADS-box protein AGL24	4.10	0.00198
MDP0000127499	AT4G17750	Heat stress transcription factor A-1a	11.39	0.00309
MDP0000301101	AT5G16820	Heat stress transcription factor A-1b	2.34	0.00047
MDP0000260377	AT5G16820	Heat stress transcription factor A-1b	9.80	1.52E-64
MDP0000174161	AT5G03720	Heat shock transcription factor A3	6.00	0.00315
MDP0000925901	AT3G22830	Heat stress transcription factor A-6b	35.21	8.90E-18
MDP0000119199	AT3G22830	Heat stress transcription factor A-6b	10.40	1.88E-07
MDP0000325078	AT3G63350	Heat stress transcription factor A-7b	8.47	0.00185
MDP0000254208	AT4G36990	Heat stress transcription factor B-1	7.47	1.98E-20
MDP0000622590	AT2G41690	Heat stress transcription factor B-3	16.29	0.00508
MDP0000241173	AT1G07890	L-ascorbate peroxidase 1	6.78	9.39E-96
MDP0000199034	AT1G07890	L-ascorbate peroxidase 1	2.54	6.07E-24
MDP0000210077	AT3G09640	L-ascorbate peroxidase 2	4.04	6.32E-09
MDP0000678891	AT4G35090	Catalase-2	2.13	8.63E-10
MDP0000147628	AT1G20620	Catalase-3	2.04	2.54E-08
MDP0000511650	AT2G47730	Glutathione S-transferase (GST6)	2.65	3.20E-05
MDP0000757070	AT5G38760	Late embryogenesis abundant protein family protein	6.22	2.81E-08
MDP0000937986	AT5G52300	Low-temperature-induced 65 (RD29B)	9.67	0.00192
MDP0000268523	AT5G25610	Dehydration-responsive protein RD22	9.18	4.69E-58
MDP0000908727	AT5G15970	Stress-induced protein KIN2	2.35	2.99E-10

a *Gene locus corresponds to annotation ID from apple (Malus domestica) genome*.

b *Arabidopsis genome initiative number*.

c *Fold change means the relative gene transcript level between the transgenic apple line and wild-type samples based on three biological replicates*.

*Positive and negative values indicate up and down-regulation, respectively*.

## Discussion

Environmental stresses, such as water deficits, high salt, or extreme temperatures, have adverse impacts on plant growth and development. Plants adapt and can become tolerant to these challenging conditions through various biochemical and physiological processes. In doing so, many stress-related genes are induced and responses are activated. The PhyCys are thought to function in regulating endogenous processes and guarding against various adverse growing conditions. Here, we focused on evaluating the biological roles of *MpCYS4*, a cystatin gene isolated from *M. prunifolia*.

MpCYS4, which contains one cystatin-like domain and characteristic motifs of phytocystatins, is most homologous to AtCYS-6 (At3g12490), HvCPI-4 (AJ748344), and OC-XII (Os01g16430) within Group C of the four cystatin groups (Tan et al., 2014a). After conducting the biochemical assay using purified MpCYS4 protein, we found that it activity to inhibit the catalytic activity of papain, consistent with earlier reports about cystatins isolated from *Arabidopsis* (Zhang et al., 2008), *Hordeum vulgare* (Gaddour et al., 2001), and *Brassica oleracea* (Eason et al., 2014). Expression analysis showed a pattern of *MpCYS4* activation under water deficit, exposure to 40°C, or treatment with exogenous ABA or MV (Tan et al., 2014a), suggesting its involvement in the response to environmental stresses. Subsequently, we introduced *MpCYS4* into *Arabidopsis* and apple to characterize its biological functions in detail.

Stomata are present on the leaf surface bordered by a pair of guard cells, and allow plants to regulate gas exchange and water loss by transpiration (Xie et al., 2012). Their opening and closing is affected by environmental and internal parameters that maintain the water balance and functioning of complex signal transduction pathways (Hetherington and Woodward, 2003; Clauw et al., 2015). Stomatal closure is a key ABA-mediated process for coping with water deficits (Cutler et al., 2010; Lee et al., 2013). ABA-insensitive mutants (i.e., *abi1* and *abi2*) are very susceptible to drought because regulation of their stomatal apertures is impaired (Schroeder et al., 2001). We found that overexpression of *MpCYS4* resulted in reduced water loss and enhanced drought resistance. Furthermore, the stomatal openings of 35S:*MpCYS4* transgenic plants were smaller than those of WT under water-deficit conditions, and stomatal closure was more sensitive in 35S:*MpCYS4* plants than in the WT after ABA treatment. These phenotypes were observed both in transgenic *Arabidopsis* and apple. Therefore, during water deficits, *MpCYS4* could affect ABA-mediated stomatal closure that minimizing water loss through transpiration to cope with drought conditions.

ABA is also associated with the regulation of complex signal transduction that subsequently induces the expression of stress-responsive genes to confer plant resistance (Shinozaki and Yamaguchi-Shinozaki, 2007; Pierik and Testerink, 2014). In our experiments, upon ABA treatment, we could not find significant differences between 35S:*MpCYS4 Arabidopsis* and WT plants in endogenous ABA levels, while expression increased for genes that encode Ser/Thr PP2Cs, *ABI1, ABI2*, and *AtPP2CA*, in 35S:*MpCYS4 Arabidopsis*. These proteins belong to Group A PP2Cs and play negative roles in plant ABA signal transduction (Ma et al., 2009; Park et al., 2009).

Transcript levels of *OST1*, which encodes an SNF1-related protein kinase 2 (SnRK2)-type protein kinase participated in ABA-mediated stomatal closure (Xie et al., 2006), were also apparently higher in the 35S:*MpCYS4 Arabidopsis*. This gene activates the anion channel *SLAC1* through phosphorylation, thereby regulating guard cell turgor and stomatal apertures (Geiger et al., 2009; Lee et al., 2009). Moreover, the plasma membrane-localized NADPH oxidase, *AtrbohF*, which contributes to H_2_O_2_ generation in guard cells, is also targeted by *OST1* (Kwak et al., 2003; Sirichandra et al., 2009). Consistently, the enhanced expression of *SLAC1* and *AtrbohF* was detected in our *MpCYS4*-overexpressing *Arabidopsis*, perhaps associated with the activating of *OST1* protein kinase. ABA is considered fundamental for triggering the activity of channels or transporters on guard cell membrane, thereby decreasing turgor, inducing stomatal closure (Brodribb and McAdam, 2013), and, ultimately, elevating the synthesis of H_2_O_2_ in guard cells via NADPH oxidase, thus, increased levels of H_2_O_2_ modulate ABA-controlled closure (Pei et al., 2000; Xie et al., 2014). So, we might conclude the elevated expression of *OST1, SLAC1*, and *AtrbohF* contributes to the increased ABA-induced stomatal closure in 35S:*MpCYS4 Arabidopsis*. Furthermore, ABRE binding transcription factors *ABI5* and *ABF3*, as well as other downstream targets *RD29B, RD22, RAB18*, and *KIN2*, showed enhanced transcription in ABA-treated 35S:*MpCYS4 Arabidopsis*. Thus, the altered expression of these genes may be responsible for ABA sensitivity in transgenic plants.

In addition, the RNA-Seq data revealed that *MpCYS4* overexpression led to a comprehensive transcriptomic alteration in the transgenic apple. In particular, *MpCYS4* not only induced but also suppressed the expression levels of many genes, indicating that it has both positive and negative impacts on the transcript atlas. We believe it is worth mentioning that the number of DEGs was markedly higher under drought (1556 up-regulated and 1327 down-regulated) than under normal conditions (82 and 69, respectively). One possible explanation is that *MpCYS4* undergoes certain unidentified modifications when plants are exposed to drought, which then alters its regulatory mode in activating or suppressing genes involved in the stress signaling network. Among the DEGs in our transgenic line, many have been annotated or confirmed to be involved in stress tolerance, either regulatory genes or functional ones. Transcript accumulations of two ABA receptor genes, *PYL4* and *PYL9*, were suppressed in 35S:*MpCYS4* plants under water-deficit conditions. Meanwhile, the expression of genes encoding a group of Ser/Thr PP2Cs, such as *ABI1, ABI2*, and *HAB1*, as well as *OST1* and ABRE binding transcription factors, was enhanced in those plants. Moreover, genes encoding major abiotic stress-responsive proteins, including *DREB2A, LEA, RD29B, RD22*, and *KIN2*, were also more strongly expressed in transgenic plants than in WT under drought stress. Thus, *MpCYS4* appears to positively affect the ABA signaling pathway by the induction of multiple stress-responsive genes, consistent with the results obtained from preliminary assays with *Arabidopsis*.

In conclusion, we have demonstrated that *MpCYS4*, a cystatin gene isolated from *M. prunifolia*, functions in mediating stomatal regulation and many ABA- and stress-related genes expression in response to drought. These properties indicate *MpCYS4* as a potentially appropriate candidate to manipulate plant drought response, improve our understanding of plant defense mechanisms against environmental challenges and provide a new tool for possible genetic alterations of phytocystatins. Such findings also can be used as a foundation for investigating the pivotal roles that these genes have in plant stress responses and in multiple signal transduction pathways. Further investigations are necessary to elucidate the molecular mechanism by which *MpCYS4* interacts with stress-responsive ABA signaling transduction, and pursue its target proteins and regulatory mechanisms that support *MpCYS4*-mediated drought stress response in apple.

## Availability of supporting data

The raw sequencing data of RNA-Seq analysis has been deposited in NBCI SRA under accession number SRP095318.

## Author contributions

YT performed most of the experiments and analyzed the data; ML, YY, XS, NW, and BL provided technical assistance to YT; FM provided the critical intellectual input in design of the study and preparation of the manuscript.

### Conflict of interest statement

The authors declare that the research was conducted in the absence of any commercial or financial relationships that could be construed as a potential conflict of interest. The reviewer HS and handling Editor declared their shared affiliation, and the handling Editor states that the process nevertheless met the standards of a fair and objective review.
