# Supplementary material for: Overexpression of MpCYS4, A Phytocystatin Gene from Malus prunifolia (Willd.) Borkh., Enhances Stomatal Closure to Confer Drought Tolerance in Transgenic Arabidopsis and Apple
Source: Front Plant Sci. 2017 Jan 24;8:33. doi: 10.3389/fpls.2017.00033 (PMC5258747; doi:10.3389/fpls.2017.00033)
Supplement: Supplementary file 1 [file Table1.PDF]

Table S1. Primer sequences.

Primer name	Oligonucleotide primers	Purpose or vector
MpCYS4-XhoI-F	CGCCTCGAGATGAAATCGGAATCTTACCTC	pBI221-GFP
MpCYS4-SacI-R	ATAGAGCTCGGAGTGGTCTGCCTCCATA	
MpCYS4-EcoRV-F	CGGATATCATGCAAAAATCAACCCGAATTGGCTGCG	pET-32a
MpCYS4-XhoI-R	GCCTCGAGGGAGTGGTCTGCCTCCAT	
MpCYS4-BamHI-F	CGGGATCCATGAAATCGGAATCTTACCTCT	pBI121
MpCYS4-SacI-R	ATAGAGCTCTTAGGAGTGGTCTGCCTC	
MpCYS4-F	AGGAGGAGACACCATCCGTTACC	Semi RT-PCR
MpCYS4-R	ATTAGACTTCTGCTGGAGGGACTT	
AtActin-F	GGTAACATTGTGCTCAGTGGTGG	Semi RT-PCR
AtActin-R	AACGACCTTAATCTTCATGCTGC	
MdActin-F	TGACCGAATGAGCAAGGAAATTACT	Semi RT-PCR
MdActin-R	TACTCAGCTTTGGCAATCCACATC	
ABI1-qRT-PCR-F	GTTACTGCAATAACCAATACTC	qRT-PCR
ABI1-qRT-PCR-R	GATCATCTTCTTCTCGCTAGTAA	
ABI2-qRT-PCR-F	GAGACAAGAAGACCAGGTCG	qRT-PCR
ABI2-qRT-PCR-R	GATGAGACAAGAACCTCCTC	
PP2CA-qRT-PCR-F	GGATCCGGAGGTAACAGTGA	qRT-PCR
PP2CA-qRT-PCR-R	GCCGTCTCGTTTGGTACAAC	
OST1-qRT-PCR-F	ATGGATCGACCAGCAGTGAG	qRT-PCR
OST1-qRT-PCR-R	CATTGCGTACACAATCTCTC	
ABI5-qRT-PCR-F	TGTAATACCCGCAGCTGCAC	qRT-PCR
ABI5-qRT-PCR-R	GTGGACAACTCGGGTTCCTC	
ABF3-qRT-PCR-F	AGAACCTCAACCGGTGGAGAG	qRT-PCR
ABF3-qRT-PCR-R	GGAGTCAGATCAGGTGACATCT	
SLAC1-qRT-PCR-F	CCGGGCTCTAGCACTCACT	qRT-PCR
SLAC1-qRT-PCR-R	TCAGTGATGCGACTCTTCCTC	
AtRbohF-qRT-PCR-F	GGTCACAAATCAACGACGAGAGT	qRT-PCR
AtRbohF-qRT-PCR-R	TCTTCTGTAATTCTCCCATCTTCATTC	
RD29B-qRT-PCR-F	AATTATCAGTCCAAAGTTACTGAT	qRT-PCR
RD29B-qRT-PCR-R	TTTCTGCCCGTAAGCAGTAACA	
RD22-qRT-PCR-F	ATGGCGATTCGGCTTCCTCT	qRT-PCR
RD22-qRT-PCR-R	GTAGCTGAACCACACAACAT	
RAB18-qRT-PCR-F	CGATCCAGCAGCAGTATGAC	qRT-PCR
RAB18-qRT-PCR-R	TTCGAAGCTTAACGGCCACC	
KIN2-qRT-PCR-F	GACCAACAAGAATGCCTTCCA	qRT-PCR
KIN2-qRT-PCR-R	TCCAAACGTAGTACATCTAAAGGGA	
AtActin-qRT-PCR-F	GGTGGTTCCATTCTTGCTTCCC	qRT-PCR
AtActin-qRT-PCR-R	TCATACTCGGCCTTGGAGATCC	
MpCYS4-qRT-PCR-F	TCCGTTACCTCTTCCGATCTTG	qRT-PCR
MpCYS4-qRT-PCR-R	ACCTGCTCAGCCTTGGCG	
MdEF-1α-qRT-PCR-F	ATTCAAGTATGCCTGGGTGC	qRT-PCR
MdEF-1α-qRT-PCR-R	CAGTCAGCCTGTGATGTTCC	
