# Supplementary material for: Overexpression of MpCYS4, A Phytocystatin Gene from Malus prunifolia (Willd.) Borkh., Enhances Stomatal Closure to Confer Drought Tolerance in Transgenic Arabidopsis and Apple
Source: Front Plant Sci. 2017 Jan 24;8:33. doi: 10.3389/fpls.2017.00033 (PMC5258747; doi:10.3389/fpls.2017.00033)
Supplement: Supplementary file 2 [file Table2.PDF]

Table S2. Primers used for qRT-PCR analysis to verify the RNA-Seq results.

Genes	Gene ID	Forward Primer	Reverse Primer
PYL4	MDP0000228470	CGGCGTCGTCGCAGTACCAA	TCCTGAGTCACGGCGGAGCA
PYL9	MDP0000284624	TACATAAGGAGGCACCACAG	CACCAAGGACCAGACGAGAT
ABI1	MDP0000437033	GGGAGGAACAACAAGGGA	AAGAAATGAACGGGTGAGAT
ABI2	MDP0000231674	GACGACGAATGCCTAATT	TCTTGTGCCAGAGGAGTA
HAB1	MDP0000265371	ACCCACCTAACCAGTCAC	ACCATAATCCCATCACCT
OST1	MDP0000224969	AGCACCTGAAGTCCTATC	ACTAAGAATCCGCCCAAT
ABF3	MDP0000701734	AATGCTCAGTTGGGTAGTCC	TTCGCAGGTGAAGGCGTC
	MDP0000248567	CGAACGCTTAGTCAGAAA	AAAGTCCTCCAAAGTCATC
LEA	MDP0000757070	TGGGGGAGATGACTTGGAG	CTGCTTCAGGTGTAGAAGC
RD29B	MDP0000937986	TGTGACAGGCGGTGAAGAAAT	TCAGCGATAGCGGAAGTGG
RD22	MDP0000268523	GACATGCGTCCTGGAACAAC	ATTTCTGGCAGCTTGTTGGA
KIN2	MDP0000908727	CTACAACGCTGGAGTGGCC	AACGAGTGCAGCATGACTATT
	MdEF-1α	ATTCAAGTATGCCTGGGTGC	CAGTCAGCCTGTGATGTTCC
