# Supplementary material for: Overexpression of MpCYS4, A Phytocystatin Gene from Malus prunifolia (Willd.) Borkh., Enhances Stomatal Closure to Confer Drought Tolerance in Transgenic Arabidopsis and Apple
Source: Front Plant Sci. 2017 Jan 24;8:33. doi: 10.3389/fpls.2017.00033 (PMC5258747; doi:10.3389/fpls.2017.00033)
Supplement: Supplementary file 3 [file Table3.PDF]

Table S3. Number of reads cleaned and mapped with *Tophat2* among RNA-Seq samples.

Sample	No. Clean Reads	No. Unmapped reads	No. Unique mapped reads	No. Multiple mapped reads	Mapping Percentage (%)
C-WT-1	41,816,878	11,634,465 (27.82%)	26,767,887 (64.01%)	3,414,526 (8.17%)	72.18%
C-WT-2	42,822,850	12,105,918 (28.27%)	27,209,836 (63.54%)	3,507,096 (8.19%)	71.73%
C-WT-3	45,225,014	12,958,160 (28.65%)	28,656,212 (63.36%)	3,610,642 (7.98%)	71.35%
C-#4-1	45,899,444	12,534,774 (27.31%)	29,603,036 (64.50%)	3,761,634 (8.20%)	72.69%
C-#4-2	42,059,078	11,562,141 (27.49%)	27,090,903 (64.41%)	3,406,034 (8.10%)	72.51%
C-#4-3	67,366,104	19,767,471 (29.34%)	42,246,261 (62.71%)	5,352,372 (7.95%)	70.66%
D-WT-1	62,267,976	17,971,483 (28.86%)	39,337,195 (63.17%)	4,959,298 (7.96%)	71.14%
D-WT-2	53,787,706	15,977,134 (29.70%)	33,666,386 (62.59%)	4,144,186 (7.70%)	70.30%
D-WT-3	41,597,252	12,405,714 (29.82%)	25,962,100 (62.41%)	3,229,438 (7.76%)	70.18%
D-#4-1	74,444,102	22,689,480 (30.48%)	45,805,902 (61.53%)	5,948,720 (7.99%)	69.52%
D-#4-2	49,463,458	14,923,038 (30.17%)	30,590,026 (61.84%)	3,950,394 (7.99%)	69.83%
D-#4-3	43,266,354	13,284,995 (30.71%)	26,587,333 (61.45%)	3,394,026 (7.84%)	69.29%

Notes: C-WT-1,C-WT-2,C-WT-3,C-#4-1,C-#4-2,C-#4-3: wild-type apple and transgenic line #4 samples under normal growth conditions;D-WT-1,D-WT-2,D-WT-3,D-#4-1,D-#4-2,D-#4-3: wild-type apple and transgenic line #4 samples after drought treatment.
