# Supplementary material for: Overexpression of MpCYS4, A Phytocystatin Gene from Malus prunifolia (Willd.) Borkh., Enhances Stomatal Closure to Confer Drought Tolerance in Transgenic Arabidopsis and Apple
Source: Front Plant Sci. 2017 Jan 24;8:33. doi: 10.3389/fpls.2017.00033 (PMC5258747; doi:10.3389/fpls.2017.00033)
Supplement: Supplementary file 4 [file Table4.PDF]

Table S4. Correlation coefficients of transcriptome profiles among RNA-Seq samples.

Sample	C-WT-1	C-WT-2	C-WT-3	C-#4-1	C-#4-2	C-#4-3	D-WT-1	D-WT-2	D-WT-3	D-#4-1	D-#4-2	D-#4-3
**C-WT-1**	1	0.997660362	0.99869125	0.995904493	0.99109826	0.990192734	0.97722484	0.97633182	0.98156497	0.91944736	0.92609763	0.912525274
**C-WT-2**	0.99766036	1	0.997631371	0.994912863	0.99176444	0.993496109	0.98380524	0.98239123	0.98620051	0.91421122	0.92022299	0.905156993
**C-WT-3**	0.99869125	0.997631371	1	0.994798444	0.98915797	0.988875228	0.98018489	0.98022898	0.98334838	0.92161956	0.92845655	0.913750156
**C-#4-1**	0.99590449	0.994912863	0.994798444	1	0.99550162	0.994118011	0.97938537	0.97547515	0.98429281	0.92179974	0.93012934	0.915203867
**C-#4-2**	0.99109826	0.991764443	0.989157973	0.995501617	1	0.990356289	0.96794564	0.9625555	0.97290232	0.89565058	0.9035629	0.887578451
**C-#4-3**	0.99019273	0.993496109	0.988875228	0.994118011	0.99035629	1	0.9846998	0.97780474	0.98613311	0.92152719	0.92627867	0.911764914
**D-WT-1**	0.97722484	0.98380524	0.980184885	0.979385374	0.96794564	0.984699796	1	0.99788146	0.99796878	0.92630877	0.9318862	0.91694456
**D-WT-2**	0.97633182	0.982391234	0.980228979	0.975475146	0.9625555	0.97780474	0.99788146	1	0.99627992	0.92202407	0.92845009	0.913844323
**D-WT-3**	0.98156497	0.986200514	0.983348381	0.984292814	0.97290232	0.986133109	0.99796878	0.99627992	1	0.92365885	0.93078113	0.917399794
**D-#4-1**	0.91944736	0.914211224	0.921619555	0.921799738	0.89565058	0.921527188	0.92630877	0.92202407	0.92365885	1	0.997794	0.996018845
**D-#4-2**	0.92609763	0.920222987	0.928456552	0.930129343	0.9035629	0.926278673	0.9318862	0.92845009	0.93078113	0.997794	1	0.995099254
**D-#4-3**	0.91252527	0.905156993	0.913750156	0.915203867	0.88757845	0.911764914	0.91694456	0.91384432	0.91739979	0.99601885	0.99509925	1

Notes: C-WT-1,C-WT-2,C-WT-3,C-#4-1,C-#4-2,C-#4-3: wild-type apple and transgenic line #4 samples under normal growth conditions; D-WT-1,D-WT-2,D-WT-3,D-#4-1,D-#4-2,D-#4-3: wild-type apple and transgenic line #4 samples after drought treatment.
