# Supplementary material for: Overexpression of MpCYS4, A Phytocystatin Gene from Malus prunifolia (Willd.) Borkh., Enhances Stomatal Closure to Confer Drought Tolerance in Transgenic Arabidopsis and Apple
Source: Front Plant Sci. 2017 Jan 24;8:33. doi: 10.3389/fpls.2017.00033 (PMC5258747; doi:10.3389/fpls.2017.00033)
Supplement: Supplementary file 5 [file Image1.PDF]

Figure S1

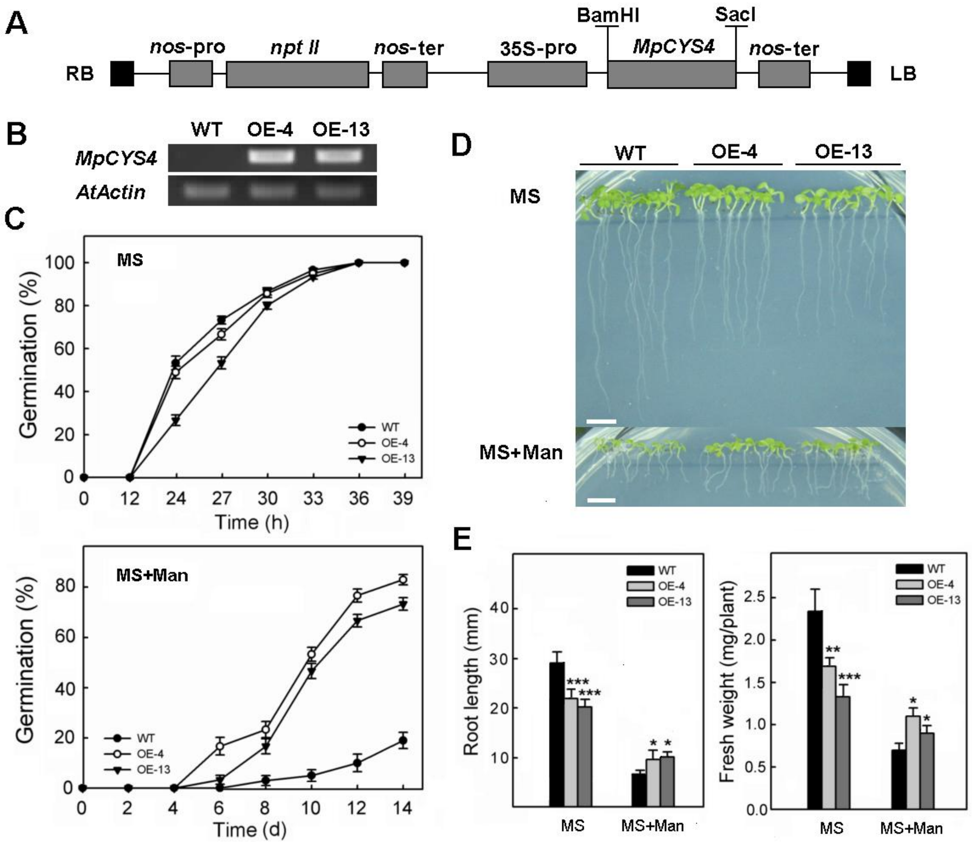

Figure S1 Effects of osmotic stress on seed germination and early seedling development of transgenic Arabidopsis plants. **(A)** Structure of 35S:*MpCYS4* construct for expression of *MpCYS4*. Gene encoding bacterial neomycin phosphotransferase II (*npt II*), which is regulated by nopaline synthase gene promoter (*nos*-pro) and 3'-terminator (*nos*-ter), served as selectable marker for *Arabidopsis* transformation. *MpCYS4* was regulated by CaMV 35S promoter (35S-pro). LB and RB indicate left and right T-DNA borders, respectively; **(B)** Expression levels of *MpCYS4* in two independent transgenic lines (OE-4, OE-13) that over-expressed *MpCYS4*. *AtActin* served as control; **(C)** Germination rate of seeds from wild-type (WT) and transgenic plants (OE-4, OE-13) sown on 1/2 MS media supplemented with 0 mM or 400 mM mannitol. Results are means ± SD from 3 independent experiments (approximately 50 seeds per line were sown for each experiment); **(D)** Comparison of seedling growth under osmotic stress. WT and transgenic plants were germinated on 1/2 MS agar plates, then transferred and vertically plated onto new 1/2 MS medium supplemented with 0 mM or 400 mM mannitol. Photo was taken after 10 days. Scale bar, 5 mm; **(E)** Root lengths and fresh weights of WT and transgenic seedlings after 10 days of treatment with 400 mM mannitol. Results are means and SDs from 3 independent experiments (approximately 10 seedlings per line per experiment). Data were significantly different from WT values at **P* <0.05, ***P* <0.01, or ****P* <0.001, based on Student's *t*-tests.
