# Supplementary material for: Overexpression of MpCYS4, A Phytocystatin Gene from Malus prunifolia (Willd.) Borkh., Enhances Stomatal Closure to Confer Drought Tolerance in Transgenic Arabidopsis and Apple
Source: Front Plant Sci. 2017 Jan 24;8:33. doi: 10.3389/fpls.2017.00033 (PMC5258747; doi:10.3389/fpls.2017.00033)
Supplement: Supplementary file 7 [file Image3.PDF]

Figure S3

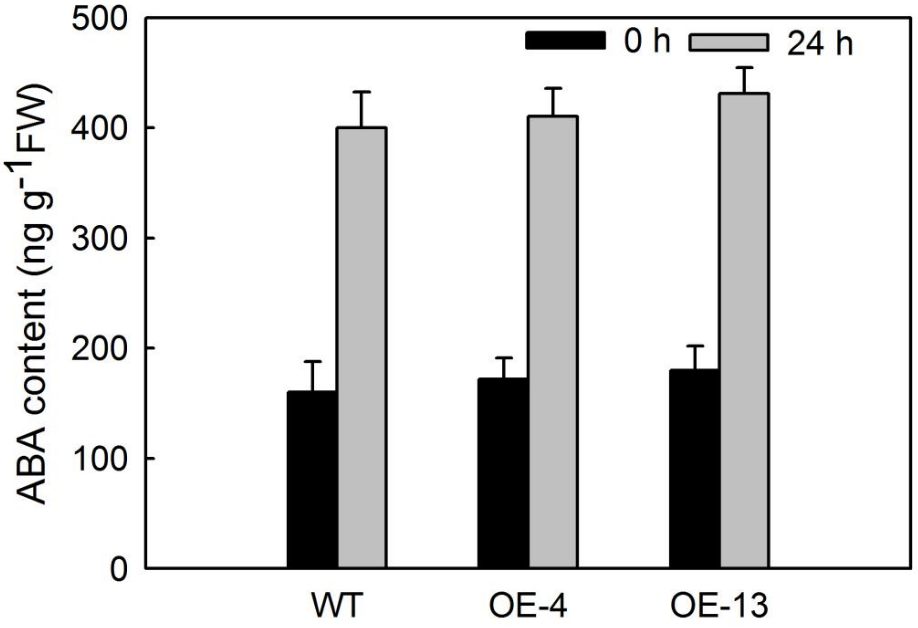

Figure S3 ABA levels in wild-type (WT) and 35S:*MpCYS4* transgenic *Arabidopsis* (lines OE-4 and OE-13) before (0 h) and after subjected to 100 μM ABA for 24 h. Data are means ± SD from 3 independent experiments.
