# Supplementary material for: Overexpression of MpCYS4, A Phytocystatin Gene from Malus prunifolia (Willd.) Borkh., Enhances Stomatal Closure to Confer Drought Tolerance in Transgenic Arabidopsis and Apple
Source: Front Plant Sci. 2017 Jan 24;8:33. doi: 10.3389/fpls.2017.00033 (PMC5258747; doi:10.3389/fpls.2017.00033)
Supplement: Supplementary file 8 [file Image4.PDF]

Figure S4

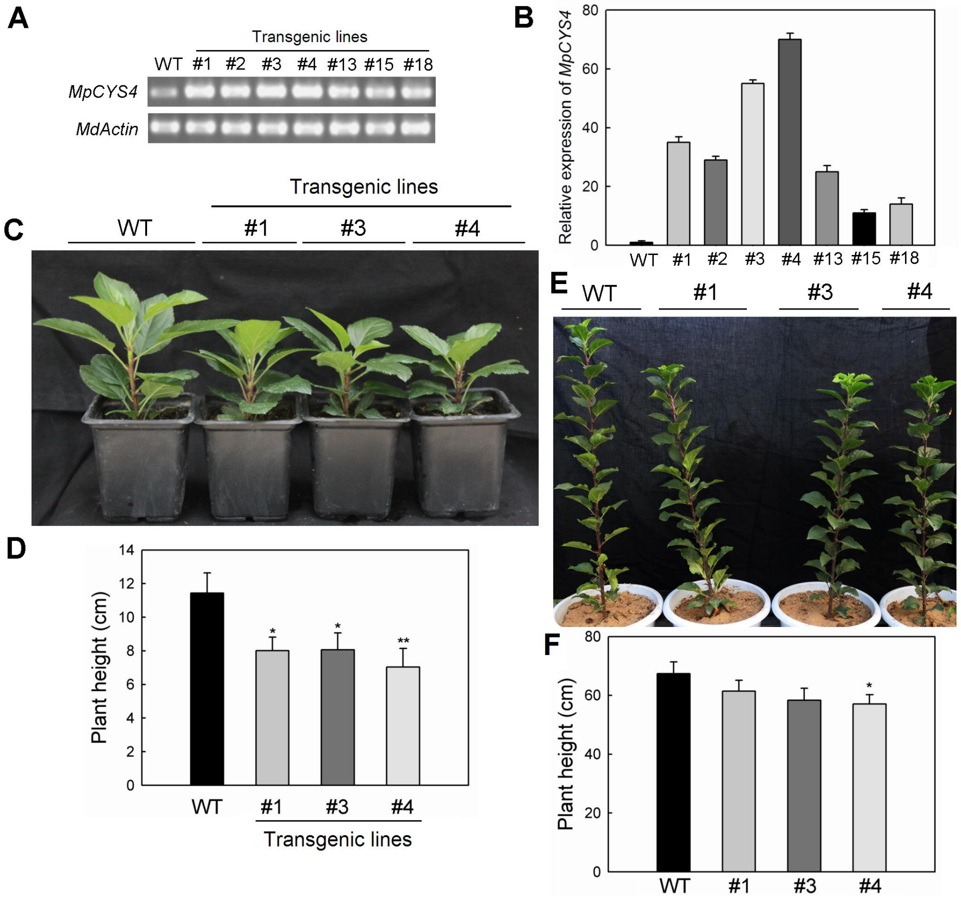

Figure S4 Generation of *MpCYS4*-overexpressing apple and phenotype analysis. **(A and B)** Semi-quantitative RT-PCR **(A)** and quantitative real-time PCR (qRT-PCR) **(B)** analysis of *MpCYS4* expression levels in independent transgenic apple lines. Data represent means ± SD of 3 independent replicates; **(C)** Phenotypes of field-grown two-month old plants regrown in pots for photography; **(D)** Measurement of plant height of **(C)**; **(E)** Phenotypes of field-grown five-month old plants were regrown in pots for photography; **(F)** Measurement of plant height of **(E)**. For D and F, each column represents an average of at least 15 plants. Error bars indicate SD. Data were significantly different from WT values at $^{*}P < 0.05$ or $^{**}P < 0.01$, based on Student's *t*-tests.
