# Supplementary material for: Overexpression of MpCYS4, A Phytocystatin Gene from Malus prunifolia (Willd.) Borkh., Enhances Stomatal Closure to Confer Drought Tolerance in Transgenic Arabidopsis and Apple
Source: Front Plant Sci. 2017 Jan 24;8:33. doi: 10.3389/fpls.2017.00033 (PMC5258747; doi:10.3389/fpls.2017.00033)
Supplement: Supplementary file 10 [file Image6.PDF]

Figure S6

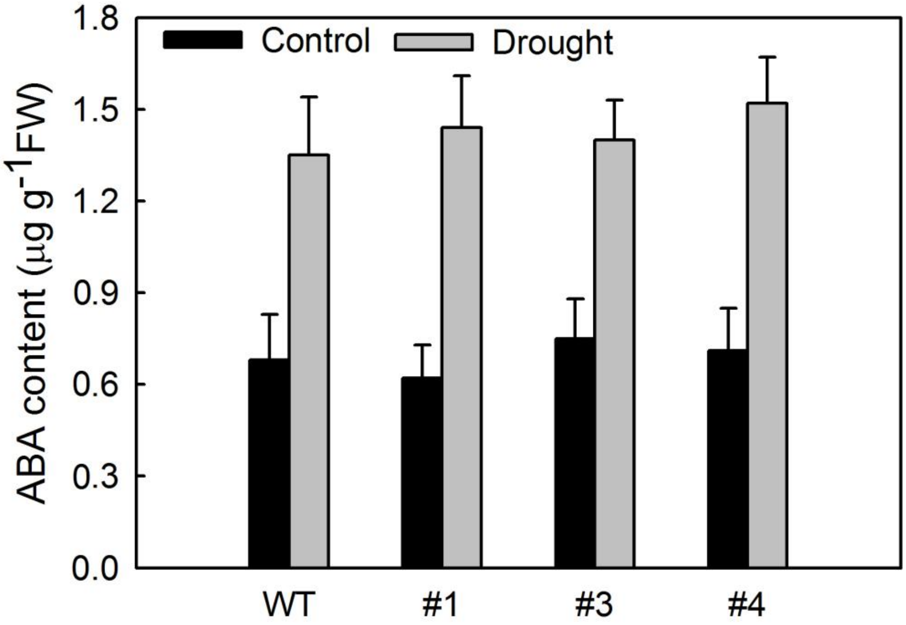

Figure S6 ABA contents in wild-type (WT) and 35S:*MpCYS4* transgenic apple lines #1, #3, and #4 under control and after water was withheld for 3 days. Data are means ± SD of 3 independent experiments.
