# Supplementary material for: Overexpression of MpCYS4, A Phytocystatin Gene from Malus prunifolia (Willd.) Borkh., Enhances Stomatal Closure to Confer Drought Tolerance in Transgenic Arabidopsis and Apple
Source: Front Plant Sci. 2017 Jan 24;8:33. doi: 10.3389/fpls.2017.00033 (PMC5258747; doi:10.3389/fpls.2017.00033)
Supplement: Supplementary file 11 [file Image7.PDF]

Figure S7

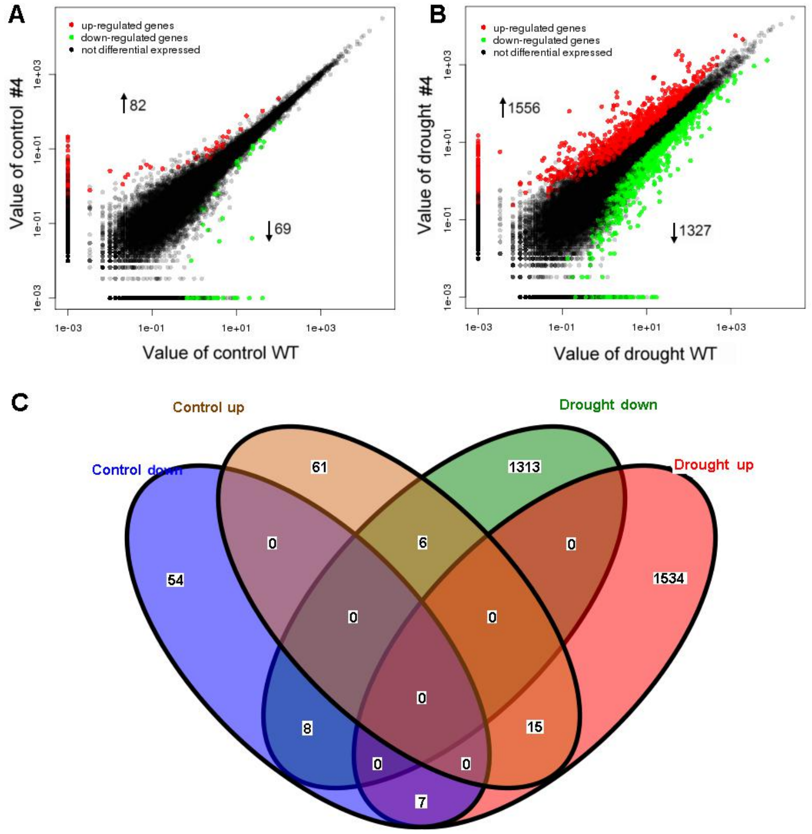

Figure S7 RNA-Seq analysis of apple wild type and transgenic line #4. Three independent biological replicates were collected for each of the genotypes, wild type (WT) or #4, that were grown under normal growth conditions or after drought treatment for 3 days. **(A and B)** Scatterplots of the expression patterns of the genes in the transgenic line (#4) compared with the wild type (WT) before **(A)** and after **(B)** drought treatment. ↑ and ↓, followed by numbers, mean the number of genes up-regulated and down-regulated in #4 compared with WT (2-fold change); **(C)** Venn diagram indicating the numbers of genes with altered transcript levels in the transgenic line compared with WT before and after drought stress.
